# A proteo-transcriptomic map of non-alcoholic fatty liver disease signatures

**DOI:** 10.1038/s42255-023-00775-1

**Published:** 2023-04-10

**Authors:** Olivier Govaere, Megan Hasoon, Leigh Alexander, Simon Cockell, Dina Tiniakos, Mattias Ekstedt, Jörn M. Schattenberg, Jerome Boursier, Elisabetta Bugianesi, Vlad Ratziu, LITMUS Investigators, Ann K. Daly, Quentin M. Anstee

**Affiliations:** 1 Translational and Clinical Research Institute, Faculty of Medical Sciences, Newcastle University, Newcastle upon Tyne, UK; 2 Department of Imaging and Pathology, KU Leuven and University Hospitals Leuven, Leuven, Belgium; 3 Bioinformatics Support Unit, Faculty of Medical Sciences, Newcastle University, Newcastle upon Tyne, UK; 4 SomaLogic, Inc., Boulder, CO USA; 5 Department of Pathology, Aretaieio Hospital, National and Kapodistrian University of Athens, Athens, Greece; 6 Department of Health, Medicine and Caring Sciences, Linköping University, Linköping, Sweden; 7 Department of Medicine, University Hospital Mainz, Mainz, Germany; 8 Hepatology Department, Angers University Hospital, Angers, France; 9 Department of Medical Sciences, Division of Gastro-Hepatology, City of Health and Science of Turin, University of Turin, Turin, Italy; 10 Assistance Publique-Hôpitaux de Paris, Hôpital Pitié Salpêtrière, Sorbonne University, ICAN (Institute of Cardiometabolism and Nutrition), Paris, France; 11 Newcastle NIHR Biomedical Research Centre, Newcastle upon Tyne Hospitals NHS Trust, Newcastle upon Tyne, UK

**Keywords:** Non-alcoholic fatty liver disease, Biomarkers, Metabolism

## Abstract

Non-alcoholic fatty liver disease (NAFLD) is a common, progressive liver disease strongly associated with the metabolic syndrome. It is unclear how progression of NAFLD towards cirrhosis translates into systematic changes in circulating proteins. Here, we provide a detailed proteo-transcriptomic map of steatohepatitis and fibrosis during progressive NAFLD. In this multicentre proteomic study, we characterize 4,730 circulating proteins in 306 patients with histologically characterized NAFLD and integrate this with transcriptomic analysis in paired liver tissue. We identify circulating proteomic signatures for active steatohepatitis and advanced fibrosis, and correlate these with hepatic transcriptomics to develop a proteo-transcriptomic signature of 31 markers. Deconvolution of this signature by single-cell RNA sequencing reveals the hepatic cell types likely to contribute to proteomic changes with disease progression. As an exemplar of use as a non-invasive diagnostic, logistic regression establishes a composite model comprising four proteins (ADAMTSL2, AKR1B10, CFHR4 and TREM2), body mass index and type 2 diabetes mellitus status, to identify at-risk steatohepatitis.

## Abstract (web summary)

Govaere et al. integrate circulating protein data from more than 300 patients with non-alcoholic fatty liver disease (NAFLD) with transcriptomics and develop a non-invasive diagnostics tool to identify patients with at-risk NAFLD based on body mass index, type 2 diabetes status and four circulating proteins.

## Main

Non-alcoholic fatty liver disease (NAFLD) is a chronic, progressive condition affecting about 25% of the global population that is strongly associated with features of the metabolic syndrome, including obesity and type 2 diabetes mellitus (T2DM)^1^. NAFLD is characterized by excessive accumulation of hepatic triglyceride and encompasses a range of disease states: from steatosis (non-alcoholic fatty liver, NAFL) through non-alcoholic steatohepatitis (NASH), defined by the presence of hepatocyte ballooning and lobular inflammation with increasing fibrosis stage, to cirrhosis and hepatocellular carcinoma^1^. Not every patient diagnosed with NAFL will develop NASH or progress to cirrhosis and end-stage liver disease, meaning that there is substantial interindividual variation in disease severity. Patients with greater steatohepatitic disease activity, defined by a histological NAFLD Activity Score (NAS, the sum of steatosis, hepatocyte ballooning and lobular inflammation) more than or equal to 4 with fibrosis stage of 2 or more (*F* ≥ 2) are considered to show ‘at-risk NASH’ that indicates a high likelihood of progressive disease^2,3^.

Several non-invasive tests have been developed to identify patients with advanced liver fibrosis. These include use of indirect markers reflecting liver function and biochemical changes, such as the NAFLD Fibrosis Score (NFS) or the FIB-4 (ref. ^4^), and biomarkers that directly measure collagen turnover, including cleaved pro-collagen type 3 peptide^5^ or thrombospondin-2 (ref. ^6^). The FibroScan-AST (FAST) score based on imaging assessment has proved to be an efficient way to identify NASH patients considered to be at risk of progressive disease^7^. More recently, proteomic approaches have been used to identify classifiers that differentiate advanced from early fibrosis^8,9^. In contrast, effective biomarkers that identify steatohepatitis and grade activity remain elusive, the field therefore relies on histological assessment that is invasive and has considerable interobserver variability.

**Fig. 1:**
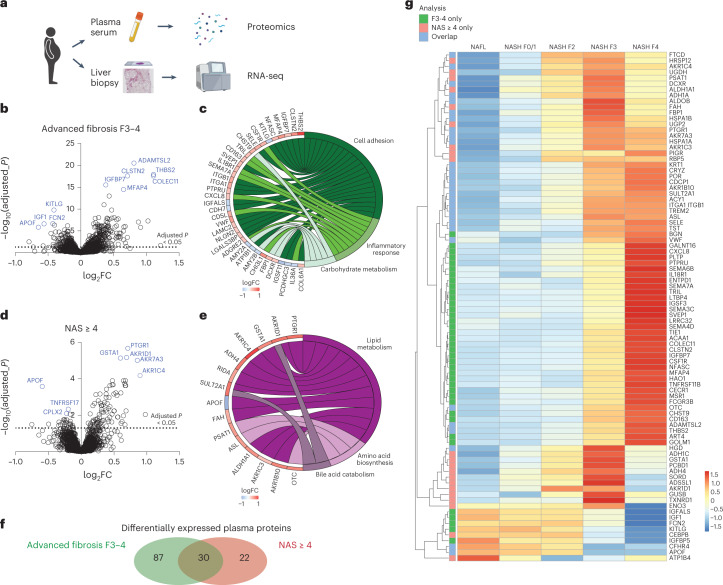
Proteomics analysis from patients with histologically proven NAFLD to identify circulating markers. **a**, Schematic overview of the study. **b**,**c**, Differentially expressed proteins in the discovery cohort of 191 patients stratified on the basis of fibrosis stage F3–4 versus baseline F0–2 (Benjamini–Hochberg false discovery rate) (**b**) and functional annotation enrichment analysis (**c**). **d**,**e**, Differentially expressed proteins in 191 patients stratified on the basis of a high disease activity score NAS ≥ 4 (Benjamini–Hochberg false discovery rate) (**d**) and enrichment analysis (**e**). **f**, Venn diagram showing overlap between the two different analyses. **g**, Heatmap showing expression of top 50 most significant proteins associated with fibrosis and NAS ≥ 4. FC, fold change. Source data

**Extended Data Fig. 1:**
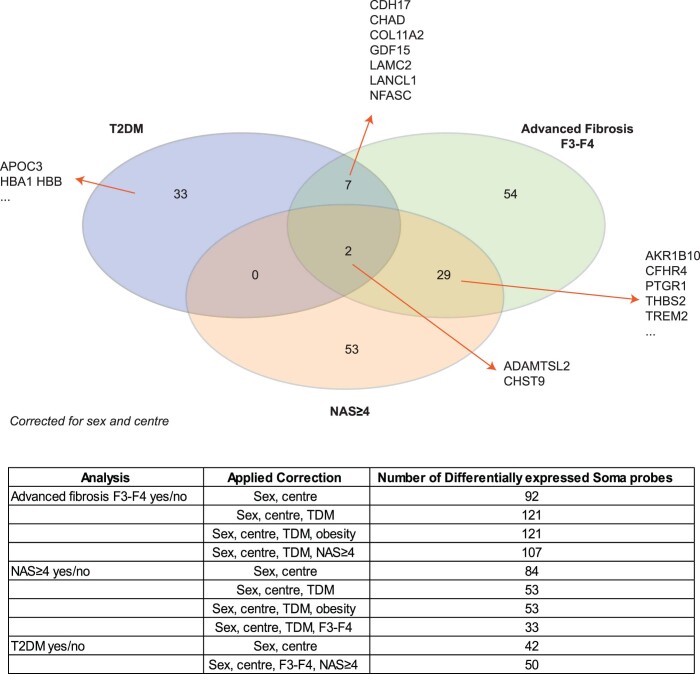
Exploration of confounding effects on the expression of Soma probes. Differentially expressed Soma probes after correction for sex and centre in the discovery cohort (n = 191) stratified based on fibrosis, NAS ≥ 4 and T2DM. The lower panel provides an overview showing the effect of corrections on the probe expression.

In this study, we integrate proteomics and RNA sequencing (RNA-seq) approaches to understand pathophysiological changes associated with NAFLD in humans and establish whether candidate circulating biomarkers might originate from the liver (Fig. 1a); a similar approach to that used recently in human alcoholic liver disease^10^ and NAFLD animal models^11^. Our study included 336 samples from patients with histologically characterized NAFLD derived from the European NAFLD Registry^12^. The discovery cohort comprised 191 plasma samples and the independent validation cohorts included 115 serum samples together with 30 liver biopsies. Within the discovery cohort, 38.4% were female, the average age was 55.2 (±11) years, average body mass index (BMI) was 33.5 (±6.7) and 60.7% had type 2 diabetes (Table 1 and Supplementary Table 1). Samples were processed for proteomics using the SomaScan v.4.0 platform, measuring 4,730 unique proteins and reads were corrected for sex, centre and T2DM (Extended Data Fig. 1). When stratifying patients on the basis of fibrosis stage (F), ranging from 0 to 4 and comparing advanced (F3–4) with mild (F0–2), we found 117 unique proteins (121 probes) to be differentially expressed (Fig. 1b and Supplementary Table 2). Functional annotation enrichment clustered proteins correlating to pathways such as ‘cell adhesion’, ‘inflammatory response’ and ‘carbohydrate metabolism’ (Fig. 1c). When stratifying patients on the basis of a high disease activity using NAS ≥ 4, we found 52 differentially expressed proteins (53 probes) (Fig. 1d and Supplementary Table 3). Enrichment analysis grouped proteins relating to ‘lipid metabolism’, ‘amino-acid biosynthesis’ or ‘bile acid catabolism’ (Fig. 1e). The two comparisons, advanced fibrosis and NAS ≥ 4, shared 30 differentially expressed proteins (Fig. 1f). When looking at the top 50 most significant differentially expressed proteins for each of these two comparisons, different dynamic expression patterns were observed as NAFLD progressed (Fig. 1g). Clear differences were seen in proteins associated with fibrogenesis and steatohepatitis during the pathogenesis of NAFLD: proteins associated with steatohepatitic activity (NAS ≥ 4) tended to peak in NASH F2–3 and then fall with progression to cirrhosis. By contrast, proteins purely associated with fibrosis increased steadily, peaking in cirrhosis (F4) (Fig. 1g).

**Table 1:** Patient demographics

Clinical feature	Discovery cohort (*n* = 191)	Discovery RNA-seq cohort (*n* = 52)	Validation cohort (*n* = 115)
Age (years)	55.24 ± 11.04	54.02 ± 13.25	52.32 ± 12.53
Sex (% female)	38.7	36.5	44.3
BMI	33.47 ± 6.67	31.31 ± 5.38	32 ± 5.82
T2DM (%)	60.73	57.7	52.2
Platelets ×10^9^	224.08 ± 67.57	216.1 ± 60.59	232.75 ± 62.86
Albumin (g dl^−1^)	4.47 ± 0.34	4.47 ± 0.3	4.24 ± 0.37
AST (μ l^−1^)	43.27 ± 21.80	42.44 ± 24.31	49.82 ± 31.37
ALT (μ l^−1^)	59.70 ± 33.47	59.65 ± 33.03	72.39 ± 49.81
Steatosis			
1	72	19	50
2	82	27	46
3	37	6	19
Ballooning			
0	26	9	41
1	108	31	47
2	57	12	27
Lobular inflammation			
0	19	3	28
1	109	39	60
2	61	9	24
3	2	1	3
Brunt fibrosis			
0	30	11	22
1	56	14	29
2	26	7	33
3	56	12	19
4	23	8	12

To establish that the circulating proteins were of hepatic origin, and to further characterize their cellular origins within the liver, we conducted a two-stage analysis. First, a proteo-transcriptomic comparison in a cohort of matching plasma-liver biopsy samples that were a subset of the discovery cohort, and second, an integrated single-cell RNA-seq and tissue expression analysis using publicly available data^13^.

**Fig. 2:**
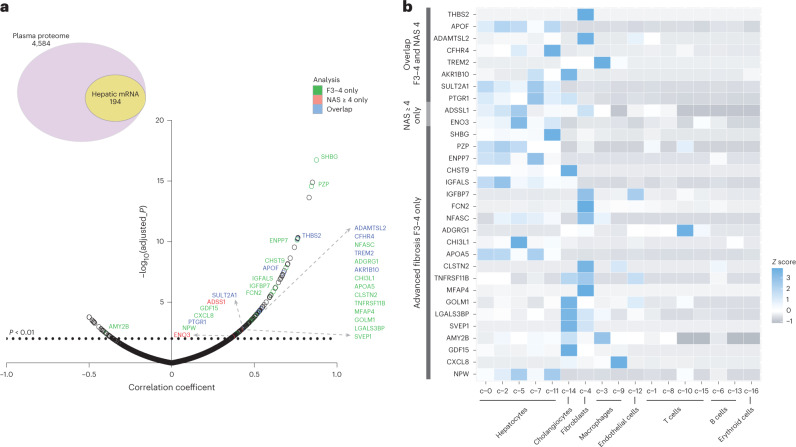
Proteo-transcriptomics correlation. **a**, Pearson correlation analysis between plasma proteome (4,584 protein probes, matching 4,292 proteins and/or genes) and matching hepatic mRNA in a subset of the discovery cohort (*n* = 52). **b**, Integrated single-cell RNA-seq analysis to deconvolute the 31 signatures of proteins associated with hepatic mRNA.

We performed linear correlations between circulating proteins on the basis of the SomaScan read-out and hepatic messenger RNA obtained from RNA-seq analysis in a subset of 52 cases from the discovery cohort with matching plasma-liver biopsy samples. Here, 4,584 protein probes, matching 4,292 proteins and/or genes, were identified in the RNA-seq data, of which 194 significantly correlated with each other (Fig. 2a and Supplementary Table 4). Within these 194 correlations, 31 proteins had been identified in the two previous comparisons described above (Fig. 2a). Eight of these 31 signals were associated with both NAS ≥ 4 and advanced fibrosis (F3–4), including THBS2, APOF, ADAMTSL2, CFHR4, TREM2, AKR1B10, SULT2A1 and PTGR1. In addition, 21 positive correlations were uniquely identified in the advanced fibrosis comparison, including GDF15, IGFBP7 and SHBG, while two correlations were from the NAS ≥ 4 comparison (ADSSL1 and ENO3) (Fig. 2a).

**Extended Data Fig. 2:**
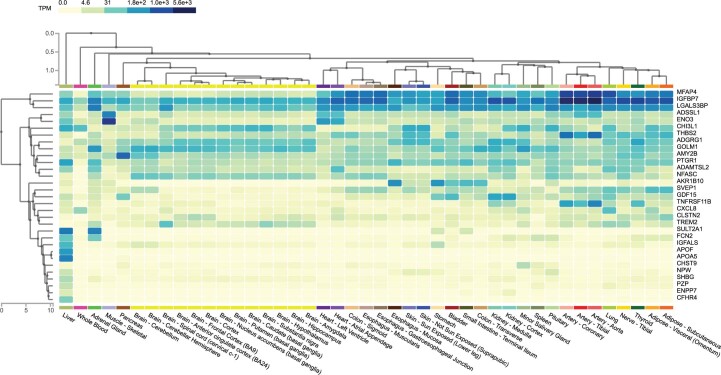
Tissue expression analysis of the 31 proteo-transcriptome signature based on GTEx portal. Expression of the hepatic 31 proteo-transcriptome signature in different organs based on GTEx portal.

GTex tissue expression analysis indicated that several of the 31 proteins in the signature are enriched in normal human liver, including the markers APOF, CFHR4, PTGR1, SULT2A1 and SHBG (Extended Data Fig. 2). Additionally, supervised analysis using bulk RNA-seq data from a large cohort of 206 patients with NAFLD^14^, showed that most of our signature changes occur in patients with advanced fibrosis and/or NAS ≥ 4 (Supplementary Table 5). Integrated single-cell RNA-seq analysis showed that the 31 signature proteins can be found in different hepatic cell populations (Fig. 2b). Of the 31 markers, 18 were enriched in epithelial cells, hepatocytes or cholangiocytes (including *AKR1B10*, *CFHR4* and *PTGR1*) compared to other hepatic cells, while other markers were primarily restricted to fibroblasts (*ADAMTSL2*, *THBS2*) or macrophages (*CXCL8* and *TREM2*) (Fig. 2b).

**Fig. 3:**
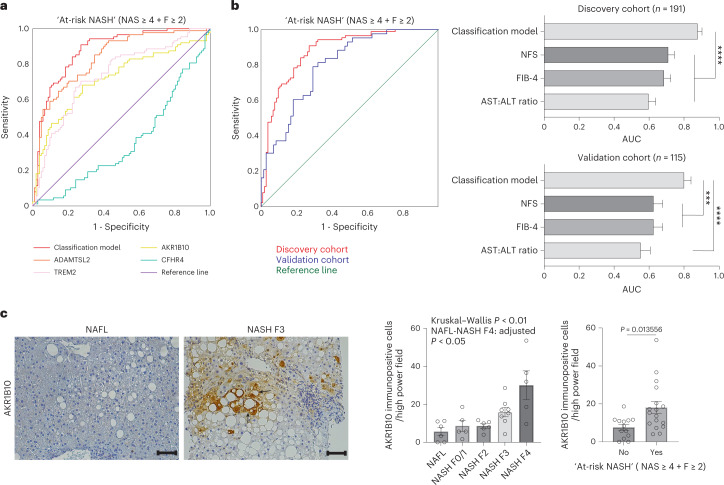
Non-invasive diagnostic tool to identify patients with at-risk NASH. **a**, Binary logistic regression modelling identified a composite model in the discovery cohort (*n* = 191 patients) that could classify patients with at-risk NASH on the basis of the variables BMI, T2DM and circulating ADAMTSL2, AKR1B10, CFHR4 and TREM2. **b**, Performance of the classification model in two independent cohorts (discovery *n* = 191 patients, validation *n* = 115 patients) in comparison with NFS, Fibrosis-4 (FIB-4) and AST:ALT ratio. Bar charts present AUC for each score with the corresponding standard error of area, as calculated by ROC analysis. Paired-sample area difference under the ROC curve test was used to compare the classification model with the other scores (discovery cohort NFS *P* = 0.000001, FIB-4 *P* = 7.8667 × 10^−7^ and AST:ALT ratio *P* = 6.85 × 10^−10^; validation cohort NFS *P* = 0.000429, FIB-4 *P* = 0.000949 and AST:ALT ratio *P* = 0.000036) (****P < 0.0001, ***P < 0.001). **c**, Representative images of immunohistochemistry and quantification for AKR1B10 in human liver biopsies (*n* = 30 biologically independent patient samples). Scale bars, 100 μm. Data are presented as mean ± s.e.m. (Kruskal–Wallis with Bonferroni correction and Mann–Whitney *U*-test). Arrows indicate necro-inflammatory region with ballooned hepatocytes. Source data

**Extended Data Fig. 3:**
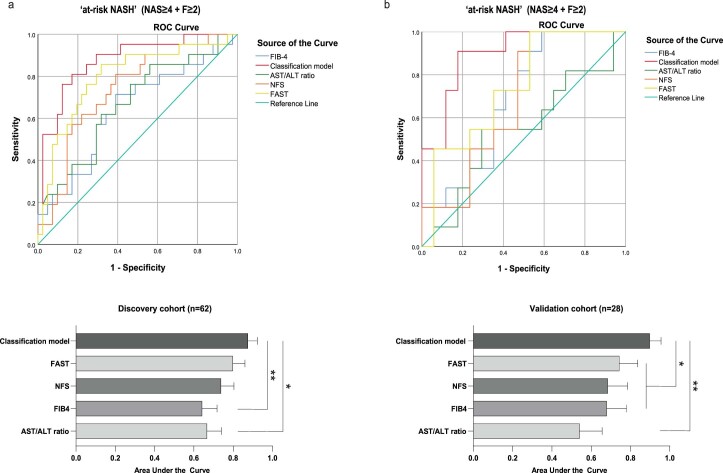
Performance of non-invasive scores to identify patients with NASH + F ≥ 2 + NAS ≥ 4. Comparison of the classification model, FibroScan-AST (FAST), NAFLD fibrosis score (NFS), Fibrosis-4 (FIB-4) and AST/ALT ratio in a subset of the discovery, n = 62 (a), and validation, n = 28 (b), cohort. Bar charts present Area under the Curve (AUC) for each score with the corresponding standard error of area, as calculated by Receiver operating characteristic (ROC) analysis. Paired-Sample Area Difference under the ROC curve test was used to compare the classification model with other scores (discovery cohort FAST p = 0.111826, NFS p = 0.050406, FIB-4 p = 0.003247 and AST/ALT ratio p = 0.016038; validation cohort FAST p = 0.045552, NFS p = 0.038341, FIB-4 p = 0.036835 and AST/ALT ratio p = 0.005652) (**p < 0.01, *p < 0.05).

To demonstrate the potential power of our proteo-transcriptomics signature strategy to support development of new non-invasive diagnostics to detect fibrosing-steatohepatitis, we performed logistic regression analysis to identify patients with ‘at-risk NASH’, defined as NAS ≥ 4 (with at least one point deriving from each NAS component) plus *F* ≥ 2 fibrosis. Backward elimination of variables identified a composite model in the discovery cohort (*n* = 191) that could classify patients with at-risk NASH with an area under the curve (AUC) of 0.878 (±0.025) including the variables BMI, T2DM and circulating ADAMTSL2, AKR1B10, CFHR4 and TREM2 (Fig. 3a), independent from any other clinical variables. The classification model had a positive predictive value of 0.79 and a negative predictive value of 0.85 (Supplementary Table 6). It significantly outperformed established non-invasive tests including the FIB-4, NFS and aspartate transaminase (AST) to alanine transaminase (ALT) ratio scores in the entire discovery cohort of 191 patients, and had a higher AUC compared to the FAST score, which was available for a subset of 62 patients (Fig. 3b, Extended Data Fig. 3 and Supplementary Table 6). These findings were validated in an independent cohort of 115 samples where the model had an AUC of 0.80 (±0.04) (Fig. 3c, Extended Data Fig. 3 and Supplementary Table 6).

In this study, we have identified proteo-transcriptomic connections associated with features of progressive NAFLD. While only CFHR4 is uniquely expressed in healthy liver (Extended Data Fig. 2), ADAMTSL2, AKR1B10 and TREM2 have been previously been reported to play a role in the progression of liver diseases and NAFLD. Single-cell RNA-seq has showed that TREM2-positive macrophages are associated with hepatic portal fibrosis, while ADAMTSL2 reflects a zonal activation of hepatic stellate cells^15,16^. Soluble ADAMTSL2 proved to be a good biomarker to identify significant and advanced fibrosis in patients with NAFLD, while circulating TREM2 levels have proved to stratify patients with NASH^9,17^. Soluble levels of TREM2 are believed to reflect the recruitment and expansion of TREM2-positive macrophages localizing to fibrotic areas in the liver, in a response to resolve steatohepatitis^18^. Using a high-throughput RNA-seq approach in a cohort of 206 NAFLD biopsies to understand the pathogenesis disease progression, we recently showed that changes in transcription of the epithelial markers *AKR1B10* and *GDF15* can also lead to altered circulating concentrations of these proteins, serving as putative biomarkers for fibrosing-steatohepatitis^14^. To support these findings, we performed immunohistochemical stainings on series of 30 NAFLD biopsies. AKR1B10 positivity was more prominent in advanced NAFLD, and was observed in ballooned hepatocytes and hepatocytes neighbouring necro-inflammatory foci and periportal/periseptal areas (Fig. 2d).

This study has some limitations as we assessed linear associations between protein and hepatic mRNA in a European White cohort only, which does not exclude the potential contribution of other organs to the expression of the circulating proteins or that other factors contribute in different ethnic groups. We were also limited in our ability to confirm some proteomic findings in hepatic tissue due to limited availability of appropriate antibodies. Nevertheless, we have highlighted the complexity of the different liver cell populations and showed that circulating proteins correlating with hepatic mRNA can be used to identify patients with at-risk NASH.

## Methods

### Patient selection

A total of 336 histologically characterized cases were derived from the European NAFLD Registry (NCT04442334); samples were collected as previously described^12^. European White patients have been treated and diagnosed for NAFLD on the basis of histology at specialized centres including Angers and Paris (France), Mainz (Germany), Turin (Italy), Linköping (Sweden) and Newcastle upon Tyne (UK). The discovery cohort comprised 191 plasma samples and the independent validation cohorts included 115 serum samples and 30 paraffin liver biopsies sections (Table 1 and Supplementary Table 1). A subset of the discovery cohort, comprising 52 of these cases had frozen liver tissue available for RNA extraction. All liver samples were centrally scored according to the semiquantitative NASH-CRN Scoring System by an expert liver pathologist. Fibrosis stage ranged from F0 to F4 (cirrhosis) and the NAS was defined as the sum of the scores for steatosis, hepatocyte ballooning and lobular inflammation^3^. Alternative diagnoses and aetiologies such as excessive alcohol intake, autoimmune liver diseases, viral hepatitis and steatogenic medication use were excluded. Sex and/or gender of participants was determined on the basis of self-report. This study has been approved by the relevant Ethical Committees in the participating centres and all patients having provided informed consent.

### Proteomics

The proteomic aptamer-based SomaScan Platform (SomaLogic) was used to process 191 plasma and 115 serum human samples (20 μl, 1 in 20 dilution)^19^. To each sample slow off-rate modified labelled aptamers were added to form SOMAmer–protein bead complexes. The beads were captured, and non-specifically bound reagents were subsequently removed. SOMAmers were quantified by hybridization to DNA microarrays. Relative quantity of SOMAmer reagents measured by the SomaScan assay reflecting original protein concentrations (that is, relative fluorescent units, RFUs). Counts were analysed for differential expression using linear models as implemented by the R package limma (https://www.bioconductor.org/) and correcting for centre, sex and T2DM. Statistical significance was determined by a corrected *P* value less than 0.05 (Benjamini–Hochberg false discovery rate) and a fold change of more than 1.25.

### RNA-seq

As previously described, mRNA was extracted from frozen liver biopsy samples and processed with the TruSeq RNA Library Prep Kit v.2 and sequenced on the NextSeq 550 System (Illumina)^14^. Data are available on the NCBI GEO repository (GSE135251). Raw sequencing quality assessment and alignment to the reference genome (GRCh38, Ensembl release 76) was done using Fastqc (v.0.11.5) and MultiQC (v.1.2dev), and gene count tables were produced with HT-Seq. Counts were normalized using the trimmed mean of *M* values method and transformed using limma’s voom methodology. A correction for centre, sex and batch was implemented. Pearson correlation was used to investigate linearity between hepatic mRNA and circulating proteins. A *P* < 0.01 was considered significant. Tissue expression analysis was conducted using GTEx (https://gtexportal.org/). Supervised analysis was done as previously described^14^. Deconvolution to identify cell of origin was done using publicly available single-cell RNA-seq data (GSE115469) from liver samples obtained from neurological deceased individuals^13^. The transformed normalized and cluster identifiers were obtained from the Human Protein Atlas (https://www.proteinatlas.org/). For each marker of interest, the *Z* score was calculated to visualize expression per cell cluster. The DAVID annotation tool was used for functional protein pathway enrichment on the basis of UniprotKB Keywords and Homo Sapiens background^20^.

### Immunohistochemistry

Human formalin-fixed paraffin-embedded liver biopsies (*n* = 30) were immunostained with antibody directed against human AKR1B10 (ab232623, Abcam; EDTA, 1/500). Immunostains were performed manually at room temperature using Envision Flex+ reagent (Dako) as secondary antibody with 3′,3′-diaminobenzidine visualization. Immunopositive cells were quantified in three different high power fields (magnification ×400) using a bright field microscope.

### Statistics

The Kolmogorov–Smirnov and Shapiro–Wilk normality tests, one-way analysis of variance with Dunnett’s test, Chi-square, Mann–Whitney *U*-test and Kruskal–Wallis test with Bonferroni correction were performed in IBM SPSS v.s27 or GraphPad Prism 9. Binary logistic regression analysis was carried out in IBM SPSS v.27 using Backward Stepwise Likelihood Ratio model including clinical parameters sex, age, BMI, ALT, AST, albumin, platelet count and T2DM, and the uncorrected values of the circulating proteins as measured by SomaScan identified as hepatic markers associated with F3–4 and NAS ≥ 4. The model identifying patients with NASH + F ≥ 2 + NAS ≥ 4 with at least one point deriving from each NAS component, and the FIB-4, NFS and FAST scores were calculated as follows:

1. Classification model = −6.236112 + (0.082163 × BMI) + (1.110341 × T2DM) + (0.001084 × ADAMTSL2) − (0.000031 × CFHR4) + (0.000060 × TREM2) + (0.000048 × AKR1B10)
2. FIB-4^4^ = (age (years) × AST (U l^−1^))/((platelets (10^9^ per l)) × √ALT (U l^−1^))
3. NFS^4^ = 1.675 + 0.037 × age (years) + 0.094 × BMI (kg m^−^^2^) + 1.13 × T2DM + (0.99 × AST:ALT ratio) (0.013 × platelet (10^9^ per l)) (0.66 × albumin (g dl^−1^))
4. FAST^7^ = (e $${}^{(-1.65+1.07\times {\mathrm{In(LSM)}}+2.66\times 10^{-8}\, {\mathrm{CAP}}3-63.3\times {\mathrm{AST}}^{-1})}$$)/(1 + e$${}^{(-1.65+1.07\times {\mathrm{In(LSM)}}+2.66\times 10^{-8}\, {\mathrm{CAP}}3-63.3\times {\mathrm{AST}}^{-1})}$$)

Receiver operating characteristic (ROC) analyses and AUC calculations were performed with IBM SPSS v.27. Paired-Sample Area Difference under the ROC curve was used as statistical test. The binary cut-off for the classification model was set at greater than −0.4491 to rule in patients with NASH + F ≥ 2 + NAS ≥ 4, the FIB-4 score was set at more than 1.3, the FAST score at more than 0.67 to rule in and equal to or less than 0.35 to rule out. Graphs have been generated using R ggplot2, R pheatmap and GraphPad Prism 9. Illustrations within Fig. 1a were created with BioRender.com.

### Reporting summary

Further information on research design is available in the Nature Portfolio Reporting Summary linked to this article.

## Supplementary information

Supplementary Information Supplementary Tables 1–6, LITMUS consortium.

Reporting Summary

## Data Availability

RNA-seq data are available on the NCBI GEO repository (https://www.ncbi.nlm.nih.gov/geo/query/acc.cgi?acc=GSE135251). Source data are provided with this paper.

## Source data

Source Data Fig. 1 Proteomics data.
Source Data Fig. 3 Overview scans of the immunohistochemical stainings against AKR1B10 on 30 NAFLD biopsies.

## Code availability

The analysis investigating the differentially expressed proteins and transcripts was carried out following the vignette for the R package limma (https://bioconductor.org/packages/release/bioc/html/limma.html).
