# Supplementary material for: A proteo-transcriptomic map of non-alcoholic fatty liver disease signatures
Source: Nat Metab. 2023 Apr 10;5(4):572–8. doi: 10.1038/s42255-023-00775-1 (PMC10132975; doi:10.1038/s42255-023-00775-1)
Supplement: Supplementary file 1 — Supplementary Tables 1–6, LITMUS consortium. [file 42255_2023_775_MOESM1_ESM.pdf]

nature metabolism

Letter https://doi.org/10.1038/s42255-023-00775-1

# A proteo-transcriptomic map of non-alcoholic fatty liver disease signatures

In the format provided by the authors and unedited

**Table of content**


| | |
|---|---|
| Supplementary Table S1. Patient demographics discovery cohort | p2 |
| Supplementary Table S2. Proteomics analysis of 191 NAFLD plasma comparing fibrosis stage F3-4 vs baseline F0-2 | p3 |
| Supplementary Table S3. Proteomics analysis of 191 NAFLD plasma comparing NAS>=4 to NAS<4 | p6 |
| Supplementary Table S4. Correlation analysis of circulating proteins with hepatic mRNA | p8 |
| Supplementary Table S5. Proteo-transcriptomics signature and gene expression in cohort of 206 NAFLD patients | p13 |
| Supplementary Table S6. Performance of the proteome classification model in comparison with other scores | p14 |
| Consortium list LITMUS investigators | p15 |

**Supplementary Table S1. Patient demographics discovery cohort.**

| Clinical feature | Discovery cohort (n=191) | T2DM | | | F3-F4 | | | NAS>=4 | | | |
|---|---|---|---|---|---|---|---|---|---|---|---|
| | | yes | no | p-value | yes | no | p-value | yes | no | p-value | Test |
| Age (years) | 55.24 +/- 11.04 | 57.16 +/- 10.13 | 52.28 +/- 11.77 | 0.005194 | 56.86 +/- 10.37 | 54.1 +/- 11.39 | 0.086267 | 55.09 +/- 10.96 | 55.57 +/- 11.3 | 0.735964 | Mann-Whitney U Test |
| Sex (% female) | 38.7 | 39.66 | 37.33 | 0.747709 | 36.71 | 40.18 | 0.627849 | 42.75 | 30.00 | 0.093217 | chi-square |
| BMI | 33.47 +/- 6.67 | 34.7 +/- 6.6 | 31.56 +/- 6.35 | 0.000455 | 33.47 +/- 6.29 | 31.85 +/- 6.48 | 0.000009 | 34.32 +-/ 6.43 | 31.62 +/- 6.87 | 0.004339 | Mann-Whitney U Test |
| T2DM (%) | 60.73 | - | - | | 82.28 | 45.54 | 3.04E-07 | 67.18 | 46.67 | 0.007058 | chi-square |
| Plts *109 | 224.08 +/- 67.57 | 222.1 +/- 64.96 | 227.15 +/- 71.76 | 0.643818 | 216.71 +/- 79.28 | 229.29 +/- 57.73 | 0.117157 | 228.48 +/- 73.27 | 214.48 +/- 52.32 | 0.196513 | Mann-Whitney U Test |
| AST (u/L) | 43.27 +/- 21.80 | 45.49 +/- 22.33 | 39.83 +/- 20.63 | 0.083272 | 53.15 +/- 22.24 | 36.29 +/- 18.64 | 5.76E-09 | 48.85 +/- 22.34 | 31.08 +/- 14.53 | 5.55E-10 | Mann-Whitney U Test |
| ALT (u/L) | 59.70 +/- 33.47 | 60.84 +/- 34.29 | 57.93 +/- 32.33 | 0.490027 | 68.20 +-/ 33.58 | 53.71 +/- 32.22 | 0.000935 | 66.52 +/- 34.37 | 44.82 +/-26 | 0.000001 | Mann-Whitney U Test |
| Steatosis | | | | 0.453791 | | | 0.62781 | | | 3.91E-19 | chi-square |
| 1 | 72 | 41 | 31 | | 27 | 45 | | 21 | 51 | | |
| 2 | 82 | 54 | 28 | | 37 | 45 | | 73 | 9 | | |
| 3 | 37 | 21 | 16 | | 15 | 22 | | 37 | 0 | | |
| Ballooning | | | | 0.000077 | | | 1.31E-08 | | | 1.14E-11 | chi-square |
| 0 | 26 | 7 | 19 | | 1 | 25 | | 5 | 21 | | |
| 1 | 108 | 65 | 43 | | 38 | 70 | | 71 | 37 | | |
| 2 | 57 | 44 | 13 | | 40 | 17 | | 55 | 2 | | |
| Lobular Inflammation | | | | 0.074619 | | | 0.000319 | | | 1.15E-13 | chi-square |
| 0 | 19 | 7 | 12 | | 3 | 16 | | 1 | 18 | | |
| 1 | 109 | 66 | 43 | | 37 | 72 | | 68 | 41 | | |
| 2 | 61 | 41 | 20 | | 38 | 23 | | 60 | 1 | | |
| 3 | 2 | 2 | 0 | | 1 | 1 | | 2 | 0 | | |
| Brunt Fibrosis | | | | 8.56E-08 | | | 3.23E-40 | | | 0.000024 | chi-square |
| 0 | 30 | 7 | 23 | | 0 | 30 | | 13 | 17 | | |
| 1 | 56 | 26 | 30 | | 0 | 56 | | 30 | 26 | | |
| 2 | 26 | 18 | 8 | | 0 | 26 | | 23 | 3 | | |
| 3 | 56 | 46 | 10 | | 56 | 0 | | 47 | 9 | | |
| 4 | 23 | 19 | 4 | | 23 | 0 | | 18 | 5 | | |
| NASH (%) | 82.2 | 91.38 | 68.00 | 0.000037 | 72.32 | 96.20 | 0.000021 | 95.42 | 53.33 | 1.69E-12 | chi-square |
| NAS>=4 (%) | 68.59 | 75.86 | 57.33 | 0.007058 | 82.28 | 58.93 | 0.000617 | - | - | | chi-square |
| F3-4 (%) | 41.14 | 56.03 | 18.67 | 3.04E-07 | - | - | | 49.62 | 23.33 | 0.000617 | chi-square |

**Supplementary Table S2. Proteomics analysis of 191 NAFLD plasma comparing fibrosis stage F3-4 vs baseline F0-2, corrected for sex, centre and T2DM (Benjamini-Hochberg false discovery rate)**

| Seq_ID | Prot_ID | logFC | AveExpr | t | P.Value | adj.P.Val |
|---|---|---|---|---|---|---|
| 6379-62 | ADAMTSL2 | 0.811499 | 5.441107 | 11.94999 | 5.62E-25 | 2.96E-21 |
| 3339-33 | THBS2 | 1.110356 | 9.459685 | 10.9796 | 4.36E-22 | 1.15E-18 |
| 18882-7 | CLSTN2 | 0.713373 | 4.826478 | 10.76768 | 1.84E-21 | 2.57E-18 |
| 4430-44 | COLEC11 | 1.108784 | 7.924007 | 10.75937 | 1.95E-21 | 2.57E-18 |
| 3320-49 | IGFBP7 | 0.380736 | 10.55276 | 10.02049 | 2.78E-19 | 2.93E-16 |
| 5636-10 | MFAP4 | 0.654039 | 5.902806 | 9.632734 | 3.6E-18 | 3.17E-15 |
| 7179-69 | NFASC | 0.433402 | 5.362726 | 9.072233 | 1.37E-16 | 1.04E-13 |
| 2844-53 | TIE1 | 0.354911 | 8.133766 | 8.326696 | 1.53E-14 | 1.01E-11 |
| 9377-25 | KITLG | -0.40869 | 6.446717 | -7.83701 | 3.07E-13 | 1.67E-10 |
| 13682-47 | CSF1R | 0.471703 | 7.48062 | 7.831953 | 3.16E-13 | 1.67E-10 |
| 3470-1 | SELE | 0.508595 | 10.16049 | 7.127598 | 2E-11 | 8.81E-09 |
| 11646-4 | CHST9 | 0.472917 | 4.958641 | 7.055498 | 3.03E-11 | 1.19E-08 |
| 6527-1 | TRIL | 0.400859 | 6.811331 | 7.048804 | 3.15E-11 | 1.19E-08 |
| 16818-200 | CDCP1 | 0.57152 | 7.203658 | 6.951237 | 5.48E-11 | 1.7E-08 |
| 5028-59 | CD163 | 0.408776 | 6.976311 | 6.950896 | 5.49E-11 | 1.7E-08 |
| 3311-27 | FCGR3B | 0.43408 | 7.471102 | 6.885696 | 7.94E-11 | 2.33E-08 |
| 17456-53 | GOLM1 | 0.54749 | 7.706002 | 6.84212 | 1.01E-10 | 2.82E-08 |
| 15431-31 | OTC | 1.025538 | 7.691387 | 6.72079 | 2E-10 | 5.27E-08 |
| 13133-73 | LTBP4 | 0.388172 | 9.177252 | 6.710255 | 2.12E-10 | 5.32E-08 |
| 18173-11 | AKR7A3 | 0.879792 | 7.708759 | 6.598203 | 3.93E-10 | 9.44E-08 |
| 6576-1 | ART4 | 0.407154 | 6.324532 | 6.574332 | 4.48E-10 | 1.03E-07 |
| 13717-15 | FCN2 | -0.42638 | 9.275241 | -6.41835 | 1.05E-09 | 2.21E-07 |
| 2952-75 | IGF1 | -0.55997 | 8.143833 | -6.39075 | 1.22E-09 | 2.38E-07 |
| 5737-61 | SEMA4D | 0.361268 | 6.229998 | 6.247696 | 2.62E-09 | 4.74E-07 |
| 19581-15 | IGFBP5 | -0.40109 | 7.085282 | -6.2424 | 2.69E-09 | 4.74E-07 |
| 3284-75 | BGN | 0.371897 | 7.898085 | 6.158789 | 4.2E-09 | 6.92E-07 |
| 11109-56 | SVEP1 | 0.414864 | 6.424363 | 6.144155 | 4.53E-09 | 7.13E-07 |
| 6077-63 | CECR1 | 0.546389 | 9.469767 | 6.133105 | 4.8E-09 | 7.13E-07 |
| 11178-21 | SVEP1 | 0.42747 | 6.421544 | 6.127931 | 4.94E-09 | 7.13E-07 |
| 9715-15 | IGSF3 | 0.373138 | 4.412386 | 6.120484 | 5.13E-09 | 7.13E-07 |
| 17782-23 | ACAA1 | 0.678144 | 8.783432 | 6.06029 | 7.03E-09 | 9.28E-07 |
| 15475-4 | PLTP | 0.325208 | 8.833601 | 6.053069 | 7.3E-09 | 9.31E-07 |
| 14079-14 | IL18R1 | 0.40612 | 8.262083 | 6.016041 | 8.86E-09 | 1.04E-06 |
| 11241-8 | ASL | 0.657557 | 9.16099 | 5.981263 | 1.06E-08 | 1.22E-06 |
| 7019-13 | SEMA7A | 0.397113 | 5.93514 | 5.97212 | 1.11E-08 | 1.22E-06 |
| 3503-4 | ITGA1 ITGB1 | 0.521772 | 9.78422 | 5.972027 | 1.11E-08 | 1.22E-06 |
| 12370-30 | APOF | -0.646 | 7.753566 | -5.92775 | 1.4E-08 | 1.45E-06 |
| 8304-50 | TNFRSF11B | 0.420184 | 7.988973 | 5.927248 | 1.4E-08 | 1.45E-06 |
| 16081-38 | AKR1B10 | 0.913563 | 8.894256 | 5.903732 | 1.58E-08 | 1.61E-06 |
| 8337-65 | PTPRU | 0.391917 | 7.472262 | 5.803305 | 2.64E-08 | 2.53E-06 |
| 5121-3 | SEMA6B | 0.370983 | 5.975784 | 5.692961 | 4.61E-08 | 4.27E-06 |
| 15533-97 | MSR1 | 0.431639 | 8.681483 | 5.628836 | 6.35E-08 | 5.68E-06 |
| 8923-94 | GALNT16 | 0.373142 | 7.018788 | 5.614127 | 6.83E-08 | 5.92E-06 |

| | | | | | | |
|---|---|---|---|---|---|---|
| 3447-64 | CXCL8 | 0.346502 | 5.664041 | 5.613587 | 6.85E-08 | 5.92E-06 |
| 9931-20 | KRT1 | 0.462207 | 6.961084 | 5.537313 | 9.98E-08 | 8.36E-06 |
| 7999-23 | ENTPD1 | 0.395724 | 6.072204 | 5.478358 | 1.33E-07 | 1.08E-05 |
| 17768-50 | HAO1 | 0.651885 | 5.847291 | 5.472537 | 1.37E-07 | 1.1E-05 |
| 7551-33 | LRRC32 | 0.322405 | 6.786715 | 5.454053 | 1.5E-07 | 1.16E-05 |
| 12663-1 | TST | 0.458216 | 6.358902 | 5.417554 | 1.79E-07 | 1.35E-05 |
| 6448-36 | SEMA3C | 0.348941 | 5.115991 | 5.372088 | 2.23E-07 | 1.59E-05 |
| 6605-17 | IGFALS | -0.384 | 9.602472 | -5.34777 | 2.51E-07 | 1.74E-05 |
| 7959-34 | CDH7 | -0.33073 | 8.583032 | -5.33277 | 2.7E-07 | 1.83E-05 |
| 3293-2 | CD5L | 0.483307 | 8.509954 | 5.308513 | 3.03E-07 | 2.02E-05 |
| 10866-60 | SMEK1 | 0.341144 | 9.642082 | 5.235261 | 4.29E-07 | 2.83E-05 |
| 18901-26 | HSPA1B | 0.386688 | 8.948832 | 5.23184 | 4.37E-07 | 2.84E-05 |
| 5508-62 | CTSD | 0.42652 | 6.873863 | 5.210118 | 4.84E-07 | 3.08E-05 |
| 18188-12 | GATM | 0.590158 | 8.771284 | 5.204006 | 4.98E-07 | 3.13E-05 |
| 19617-5 | PTGR1 | 0.549043 | 10.64063 | 5.188201 | 5.36E-07 | 3.29E-05 |
| 3050-7 | VWF | 0.611651 | 9.224145 | 5.173732 | 5.74E-07 | 3.48E-05 |
| 3049-61 | PRSS1 | -0.45488 | 7.329822 | -5.14831 | 6.47E-07 | 3.77E-05 |
| 18893-26 | GPR56 | 0.413794 | 6.245587 | 5.078277 | 8.97E-07 | 5.05E-05 |
| 9580-5 | LAMC2 | 0.373289 | 6.331564 | 5.077743 | 8.99E-07 | 5.05E-05 |
| 9772-153 | NLGN2 | 0.333421 | 4.103729 | 5.023255 | 1.16E-06 | 6.36E-05 |
| 19120-33 | DAO | 0.324642 | 4.21669 | 4.995785 | 1.31E-06 | 6.99E-05 |
| 11287-14 | CYB5A | 0.366264 | 6.012359 | 4.990105 | 1.35E-06 | 7.1E-05 |
| 10803-22 | HSPA1A | 0.344874 | 7.273378 | 4.874337 | 2.28E-06 | 0.000111 |
| 10749-18 | HSPA1A | 0.366057 | 7.532547 | 4.793865 | 3.27E-06 | 0.000151 |
| 8983-7 | GOLM1 | 0.54904 | 6.740157 | 4.783226 | 3.43E-06 | 0.000157 |
| 5000-52 | LGALS3BP | 0.357532 | 8.771033 | 4.740097 | 4.15E-06 | 0.000184 |
| 9829-91 | SULT2A1 | 0.597237 | 3.314615 | 4.739584 | 4.16E-06 | 0.000184 |
| 19197-95 | ACAT1 | 0.574738 | 8.362384 | 4.737606 | 4.19E-06 | 0.000184 |
| 6359-50 | POMGNT2 | 0.326125 | 5.508461 | 4.650638 | 6.14E-06 | 0.000257 |
| 18397-5 | AKR1C4 | 0.711662 | 8.230998 | 4.598674 | 7.69E-06 | 0.000313 |
| 4546-27 | ADGRE2 | 0.3754 | 7.663681 | 4.596416 | 7.77E-06 | 0.000313 |
| 9969-8 | SLC22A16 | -0.41783 | 8.280028 | -4.57368 | 8.57E-06 | 0.00034 |
| 18185-118 | ALDOB | 0.569289 | 8.510528 | 4.559488 | 9.11E-06 | 0.000356 |
| 13492-44 | HSPA9 | 0.428561 | 7.808643 | 4.550267 | 9.48E-06 | 0.000368 |
| 2731-29 | POR | 0.433381 | 7.837501 | 4.546331 | 9.64E-06 | 0.000371 |
| 5430-66 | SIRPA | 0.536741 | 7.309928 | 4.5041 | 1.15E-05 | 0.000426 |
| 13983-27 | CRYZ | 0.601128 | 7.950211 | 4.494313 | 1.2E-05 | 0.000441 |
| 18917-53 | AMY2A | -0.39577 | 8.43842 | -4.45967 | 1.39E-05 | 0.000497 |
| 2681-23 | HGF | 0.41172 | 5.984086 | 4.436016 | 1.54E-05 | 0.000533 |
| 13392-13 | ATP1B1 | -0.34427 | 6.654994 | -4.42278 | 1.63E-05 | 0.000558 |
| 5353-89 | IL1RN | 0.330123 | 7.032045 | 4.321998 | 2.48E-05 | 0.000797 |
| 3069-52 | IGHM IGJ IGK@ IGL@ | 0.389893 | 7.599814 | 4.285678 | 2.88E-05 | 0.000904 |
| 3343-1 | ACY1 | 0.654256 | 8.123984 | 4.216201 | 3.82E-05 | 0.001146 |
| 16620-26 | LY75 | 0.334699 | 7.919238 | 4.212885 | 3.87E-05 | 0.001155 |
| 15556-49 | AMY2B | -0.33726 | 5.591009 | -4.12582 | 5.5E-05 | 0.001527 |

| | | | | | | |
|---|---|---|---|---|---|---|
| 3310-62 | FCGR2B | 0.490749 | 6.210732 | 4.122495 | 5.57E-05 | 0.001532 |
| 4374-45 | GDF15 | 0.402443 | 9.909949 | 4.11272 | 5.79E-05 | 0.001575 |
| 4234-8 | IL1RL1 | 0.322473 | 6.944597 | 3.964429 | 0.000104 | 0.002545 |
| 15363-32 | APOA5 | -0.35482 | 8.081196 | -3.95375 | 0.000108 | 0.00264 |
| 16300-4 | TREM2 | 0.354218 | 9.66672 | 3.940584 | 0.000114 | 0.002743 |
| 6471-53 | CFHR4 | -0.78972 | 9.577361 | -3.90195 | 0.000132 | 0.003093 |
| 11104-13 | CHI3L1 | 0.681666 | 8.051781 | 3.702931 | 0.000278 | 0.00576 |
| 3313-21 | FCN2 | -0.33154 | 11.64123 | -3.69323 | 0.000288 | 0.005923 |
| 4535-50 | BST1 | 0.353099 | 8.560208 | 3.673036 | 0.000311 | 0.006256 |
| 7206-20 | FBP1 | 0.41975 | 8.95545 | 3.587905 | 0.000423 | 0.007913 |
| 4929-55 | SHBG | 0.467472 | 7.481542 | 3.559987 | 0.000467 | 0.008623 |
| 2212-69 | PLAT | 0.344 | 8.226273 | 3.543018 | 0.000496 | 0.009034 |
| 6213-10 | CGB2 | 0.356431 | 6.270862 | 3.537301 | 0.000507 | 0.009125 |
| 9986-14 | NPW | 0.570931 | 7.506144 | 3.462564 | 0.000659 | 0.011116 |
| 19335-2 | HN1 | -0.42133 | 6.558972 | -3.41963 | 0.000766 | 0.012467 |
| 12627-97 | ATG7 | -0.34016 | 4.810903 | -3.32592 | 0.001056 | 0.015914 |
| 8048-9 | FTMT | -0.36616 | 7.239857 | -3.29616 | 0.001167 | 0.017109 |
| 9253-52 | ABO | 1.216839 | 9.193858 | 3.194467 | 0.001637 | 0.022499 |
| 17787-1 | ECHS1 | 0.419809 | 9.639293 | 3.182374 | 0.001704 | 0.023169 |
| 18381-16 | ALDH2 | 0.376943 | 7.066654 | 3.181302 | 0.00171 | 0.023191 |
| 4435-66 | ENPP7 | 0.477713 | 8.20253 | 3.180055 | 0.001717 | 0.023226 |
| 17758-79 | DCXR | 0.333853 | 6.820472 | 3.174597 | 0.001747 | 0.023524 |
| 8300-82 | PEX14 | 0.3354 | 4.951527 | 3.168901 | 0.00178 | 0.023724 |
| 17396-23 | ADH1A | 0.388407 | 9.117731 | 3.152978 | 0.001875 | 0.024677 |
| 10700-10 | IGSF11 | -0.57418 | 4.403175 | -3.12046 | 0.002084 | 0.026496 |
| 7859-21 | PCDHGC3 | -0.4138 | 6.087429 | -3.03689 | 0.002722 | 0.031989 |
| 14150-7 | IL36A | -0.38983 | 6.655166 | -3.02386 | 0.002836 | 0.03304 |
| 9739-4 | MSH2 | 0.340458 | 5.0584 | 3.007958 | 0.002982 | 0.034431 |
| 12394-53 | C16orf54 | 0.345842 | 4.304297 | 2.979696 | 0.003258 | 0.036968 |
| 9213-24 | FTCD | 0.696501 | 10.54269 | 2.967442 | 0.003384 | 0.037836 |
| 9832-33 | HGD | 0.326008 | 6.96329 | 2.932773 | 0.003767 | 0.041161 |
| 6580-29 | PZP | -0.72495 | 5.579797 | -2.86449 | 0.004641 | 0.04737 |
| 16828-8 | COL6A1 | 0.402965 | 8.476906 | 2.846112 | 0.004906 | 0.049122 |

**Supplementary Table S3. Proteomics analysis of 191 NAFLD plasma comparing NAS>=4 to NAS<4, corrected for sex, centre and T2DM (Benjamini-Hochberg false discovery rate)**

| Seq_ID | Prot_ID | logFC | AveExpr | t | P.Value | adj.P.Val |
|---|---|---|---|---|---|---|
| 19617-5 | PTGR1 | 0.709834 | 10.65149 | 6.597547 | 3.95E-10 | 2.08E-06 |
| 18398-1 | AKR1D1 | 0.692049 | 5.654246 | 6.25448 | 2.53E-09 | 6.67E-06 |
| 17138-8 | GSTA1 | 0.59826 | 6.716996 | 6.164578 | 4.07E-09 | 7.16E-06 |
| 18173-11 | AKR7A3 | 0.856029 | 7.661145 | 6.056098 | 7.19E-09 | 9.48E-06 |
| 18397-5 | AKR1C4 | 0.897977 | 8.215337 | 5.635281 | 6.15E-08 | 6.49E-05 |
| 11313-100 | PCBD1 | 0.475933 | 6.902215 | 5.437433 | 1.63E-07 | 0.000125 |
| 8325-37 | ADH4 | 0.675213 | 9.441038 | 5.430993 | 1.68E-07 | 0.000125 |
| 7206-20 | FBP1 | 0.645228 | 8.988682 | 5.40629 | 1.89E-07 | 0.000125 |
| 17396-23 | ADH1A | 0.664929 | 9.163206 | 5.30882 | 3.03E-07 | 0.000177 |
| 3503-4 | ITGA1 ITGB1 | 0.487272 | 9.756303 | 5.232775 | 4.35E-07 | 0.000211 |
| 14636-25 | HRSP12 | 0.472088 | 8.968137 | 5.230079 | 4.4E-07 | 0.000211 |
| 6379-62 | ADAMTSL2 | 0.438272 | 5.33044 | 5.177044 | 5.65E-07 | 0.000232 |
| 18185-118 | ALDOB | 0.671969 | 8.51564 | 5.174509 | 5.72E-07 | 0.000232 |
| 9829-91 | SULT2A1 | 0.678077 | 3.30708 | 5.157223 | 6.2E-07 | 0.000234 |
| 12370-30 | APOF | -0.59518 | 7.809011 | -5.12113 | 7.35E-07 | 0.000258 |
| 19241-31 | RBP5 | 0.384945 | 7.686203 | 5.031741 | 1.11E-06 | 0.000367 |
| 3339-33 | THBS2 | 0.605059 | 9.309428 | 4.914384 | 1.9E-06 | 0.000589 |
| 17758-79 | DCXR | 0.528901 | 6.856828 | 4.902244 | 2.01E-06 | 0.000589 |
| 18901-26 | HSPA1B | 0.379854 | 8.930734 | 4.853145 | 2.51E-06 | 0.000673 |
| 15562-24 | GUSB | 0.497407 | 7.678665 | 4.839487 | 2.66E-06 | 0.000673 |
| 9832-33 | HGD | 0.544487 | 6.992119 | 4.77752 | 3.51E-06 | 0.000806 |
| 10749-18 | HSPA1A | 0.379269 | 7.521161 | 4.713613 | 4.66E-06 | 0.001025 |
| 16818-200 | CDCP1 | 0.424651 | 7.143365 | 4.695979 | 5.04E-06 | 0.001063 |
| 3470-1 | SELE | 0.359238 | 10.10873 | 4.542707 | 9.79E-06 | 0.001913 |
| 13967-14 | TXNRD1 | 0.396184 | 7.901958 | 4.482931 | 1.26E-05 | 0.002222 |
| 10803-22 | HSPA1A | 0.332643 | 7.257642 | 4.431744 | 1.57E-05 | 0.002669 |
| 2731-29 | POR | 0.438189 | 7.822986 | 4.349006 | 2.22E-05 | 0.003341 |
| 11424-4 | FAH | 0.413774 | 8.566145 | 4.286742 | 2.87E-05 | 0.0042 |
| 9931-20 | KRT1 | 0.379708 | 6.926123 | 4.220593 | 3.75E-05 | 0.005078 |
| 3343-1 | ACY1 | 0.684509 | 8.095875 | 4.183478 | 4.36E-05 | 0.005746 |
| 18342-2 | PSAT1 | 0.503414 | 9.654546 | 4.177686 | 4.46E-05 | 0.005746 |
| 11241-8 | ASL | 0.498862 | 9.102031 | 4.168514 | 4.63E-05 | 0.00582 |
| 16300-4 | TREM2 | 0.390028 | 9.65984 | 4.126519 | 5.48E-05 | 0.006573 |
| 13939-14 | UGP2 | 0.335935 | 4.797654 | 4.107597 | 5.91E-05 | 0.00678 |
| 3216-2 | PIGR | 0.469805 | 7.562641 | 4.024141 | 8.22E-05 | 0.008671 |
| 9213-24 | FTCD | 0.978389 | 10.57104 | 4.004959 | 8.86E-05 | 0.009043 |
| 17755-5 | UGDH | 0.643968 | 9.510802 | 3.956313 | 0.000107 | 0.010652 |
| 11265-8 | ALDH1A1 | 0.558853 | 7.884879 | 3.913553 | 0.000126 | 0.012326 |
| 13983-27 | CRYZ | 0.555098 | 7.912424 | 3.898565 | 0.000134 | 0.012496 |
| 15447-45 | SORD | 0.435121 | 11.05304 | 3.895862 | 0.000135 | 0.012496 |
| 16616-137 | ENO3 | 0.400336 | 6.769721 | 3.815639 | 0.000183 | 0.016094 |
| 12663-1 | TST | 0.343984 | 6.325106 | 3.749249 | 0.000235 | 0.018472 |
| 17377-1 | AKR1C3 | 0.325198 | 6.182696 | 3.71762 | 0.000264 | 0.019877 |

| | | | | | | |
|---|---|---|---|---|---|---|
| 16081-38 | AKR1B10 | 0.622579 | 8.784872 | 3.673698 | 0.00031 | 0.021968 |
| 13998-26 | ADSSL1 | 0.419586 | 6.180073 | 3.6716 | 0.000312 | 0.021968 |
| 3050-7 | VWF | 0.466392 | 9.170541 | 3.648013 | 0.00034 | 0.023319 |
| 15675-3 | CEBPB | -0.33984 | 5.340598 | -3.63751 | 0.000354 | 0.023613 |
| 15431-31 | OTC | 0.608183 | 7.556523 | 3.570082 | 0.000451 | 0.027658 |
| 9933-49 | ATP1B4 | -0.49831 | 4.562781 | -3.52839 | 0.000523 | 0.030325 |
| 6471-53 | CFHR4 | -0.75381 | 9.618786 | -3.50797 | 0.000562 | 0.031896 |
| 15525-294 | ADH1C | 0.667872 | 8.7253 | 3.481571 | 0.000617 | 0.032881 |
| 18449-33 | PPIG | -0.34373 | 5.868784 | -3.44375 | 0.000704 | 0.036072 |
| 5102-55 | MICB | 0.384082 | 3.901227 | 3.421671 | 0.00076 | 0.038573 |

**Supplementary Table S4. Pearson Correlation analysis of circulating proteins with hepatic mRNA.**

| Seq_ID | Target | Ensembl_ID | Official_gene_name | p value | corr.coeff | 95_conf_int | 95_conf_int.1 |
|---|---|---|---|---|---|---|---|
| 4929-55 | SHBG | ENSG00000129214 | *SHBG* | 1.78E-17 | 0.876268 | 0.79300049 | 0.927396253 |
| 15395-15 | GST M1-1 | ENSG00000134184 | *GSTM1* | 1.3E-15 | 0.851284 | 0.753413109 | 0.9122534 |
| 6580-29 | Pregnancy zone protein | ENSG00000126838 | *PZP* | 2.67E-15 | 0.846576 | 0.746031835 | 0.909381085 |
| 3504-58 | LEAP-1 | ENSG00000105697 | *HAMP* | 2.27E-14 | 0.831597 | 0.722710503 | 0.9002024 |
| 7145-1 | ITIH3 | ENSG00000162267 | *ITIH3* | 4.69E-11 | 0.763398 | 0.619575237 | 0.857627716 |
| 8323-163 | TFF3 | ENSG00000160180 | *TFF3* | 5.07E-11 | 0.762557 | 0.618333775 | 0.857094558 |
| 3339-33 | TSP2 | ENSG00000186340 | *THBS2* | 5.66E-11 | 0.761361 | 0.616568015 | 0.856335353 |
| 4435-66 | ENPP7 | ENSG00000182156 | *ENPP7* | 7E-11 | 0.75903 | 0.613133366 | 0.854855619 |
| 4874-3 | Angiogenin | ENSG00000214274 | *ANG* | 2.92E-10 | 0.742664 | 0.58917403 | 0.844422774 |
| 8960-3 | LRAP | ENSG00000164308 | *ERAP2* | 2.26E-09 | 0.716896 | 0.551986859 | 0.827837191 |
| 3060-43 | C9 | ENSG00000113600 | *C9* | 6.57E-09 | 0.702246 | 0.531133563 | 0.818320615 |
| 3367-8 | FETUB | ENSG00000090512 | *FETUB* | 7.36E-09 | 0.700632 | 0.528848957 | 0.817268357 |
| 11646-4 | Carbohydrate sulfotransferase 9 | ENSG00000154080 | *CHST9* | 1.37E-08 | 0.691603 | 0.516113511 | 0.811367127 |
| 6551-94 | Vaspin | ENSG00000165953 | *SERPINA12* | 2.6E-08 | 0.681964 | 0.502601781 | 0.805039693 |
| 12370-30 | Apo F | ENSG00000175336 | *APOF* | 3.83E-08 | 0.675931 | 0.494189475 | 0.801065208 |
| 15594-47 | HTRA1 | ENSG00000166033 | *HTRA1* | 5.35E-08 | 0.670618 | 0.48680924 | 0.79755591 |
| 4721-54 | TFF3 | ENSG00000160180 | *TFF3* | 5.46E-08 | 0.670297 | 0.486364744 | 0.797343879 |
| 4337-49 | CRP | ENSG00000132693 | *CRP* | 6.78E-08 | 0.666798 | 0.481519571 | 0.795027669 |
| 8248-222 | SIG14 | ENSG00000254415 | *SIGLEC14* | 9.35E-08 | 0.661515 | 0.474225613 | 0.791523503 |
| 16900-29 | MDGA1 | ENSG00000112139 | *MDGA1* | 1.29E-07 | 0.656064 | 0.466726394 | 0.78789884 |
| 8469-41 | IGFBP-2 | ENSG00000115457 | *IGFBP2* | 2.32E-07 | 0.645962 | 0.452899796 | 0.781157056 |
| 6605-17 | IGFALS | ENSG00000099769 | *IGFALS* | 5.82E-07 | 0.629297 | 0.430293484 | 0.76996692 |
| 18895-54 | GSTM4 | ENSG00000168765 | *GSTM4* | 6.79E-07 | 0.626426 | 0.426423196 | 0.768029947 |
| 3320-49 | IGFBP-7 | ENSG00000163453 | *IGFBP7* | 1.27E-06 | 0.614275 | 0.410126032 | 0.759804512 |
| 13717-15 | FCN2 | ENSG00000160339 | *FCN2* | 2.34E-06 | 0.602088 | 0.393911294 | 0.751508057 |
| 6081-52 | PCOC2 | ENSG00000163710 | *PCOLCE2* | 2.71E-06 | 0.599013 | 0.3898397 | 0.74940686 |
| 13676-46 | Inhibin bB chain | ENSG00000163083 | *INHBB* | 3.47E-06 | 0.593869 | 0.38304822 | 0.745885843 |
| 8356-88 | NEU1 | ENSG00000101405 | *OXT* | 3.91E-06 | 0.591349 | 0.379728647 | 0.744157413 |
| 8690-25 | CAV3 | ENSG00000182533 | *CAV3* | 9.78E-06 | 0.571164 | 0.353339281 | 0.730241233 |
| 3484-6 | Angiotensinogen | ENSG00000135744 | *AGT* | 0.000016 | 0.559836 | 0.338678335 | 0.722372371 |
| 4842-62 | Glypican 3 | ENSG00000147257 | *GPC3* | 2.28E-05 | 0.551278 | 0.32767417 | 0.716400175 |
| 6379-62 | ATL2 | ENSG00000197859 | *ADAMTSL2* | 2.52E-05 | 0.548819 | 0.32452411 | 0.714680018 |
| 9748-31 | GSTM3-3 | ENSG00000134202 | *GSTM3* | 0.000026 | 0.548133 | 0.323645349 | 0.714199308 |
| 3173-49 | ASAHL | ENSG00000138744 | *NAAA* | 3.53E-05 | 0.540526 | 0.313934481 | 0.708862516 |
| 6471-53 | FHR4 | ENSG00000134365 | *CFHR4* | 3.56E-05 | 0.540307 | 0.313654882 | 0.708708185 |
| 3313-21 | FCN2 | ENSG00000160339 | *FCN2* | 4.85E-05 | 0.532445 | 0.303669776 | 0.703171761 |
| 10565-19 | SLIK3 | ENSG00000121871 | *SLITRK3* | 0.000049 | 0.532156 | 0.303303038 | 0.70296749 |
| 2925-9 | PAI-1 | ENSG00000106366 | *SERPINE1* | 5.23E-05 | 0.530498 | 0.301203803 | 0.701796958 |
| 9754-33 | Quinone reductase 2 | ENSG00000124588 | *NQO2* | 5.57E-05 | 0.528851 | 0.299120361 | 0.700633088 |
| 7179-69 | NFASC | ENSG00000163531 | *NFASC* | 6.24E-05 | 0.52586 | 0.295344067 | 0.698518084 |
| 16300-4 | TREM2 | ENSG00000095970 | *TREM2* | 6.25E-05 | 0.525831 | 0.295306511 | 0.698497015 |
| 18893-26 | GPR56 | ENSG00000205336 | *ADGRG1* | 7.34E-05 | 0.521554 | 0.289918744 | 0.695467122 |
| 11237-49 | PCOC1 | ENSG00000106333 | *PCOLCE* | 7.58E-05 | 0.5207 | 0.288844782 | 0.694861435 |
| 16081-38 | Aldose reductase-like | ENSG00000198074 | *AKR1B10* | 8.06E-05 | 0.51907 | 0.286794938 | 0.693703774 |

| | | | | | | | |
|---|---|---|---|---|---|---|---|
| 8465-52 | Cathepsin H | ENSG00000103811 | *CTSH* | 0.000105 | 0.511833 | 0.277724543 | 0.688555856 |
| 11104-13 | YKL-40 | ENSG00000133048 | *CHI3L1* | 0.000106 | 0.511516 | 0.277329131 | 0.688330493 |
| 19557-3 | KLOTB | ENSG00000134962 | *KLB* | 0.000107 | 0.511484 | 0.277288441 | 0.688307297 |
| 6364-7 | TPSNR | ENSG00000139192 | *TAPBPL* | 0.000108 | 0.51111 | 0.276821205 | 0.688040887 |
| 3449-58 | Kallistatin | ENSG00000100665 | *SERPINA4* | 0.000109 | 0.510864 | 0.276514468 | 0.68786593 |
| 13123-3 | FLRT3 | ENSG00000125848 | *FLRT3* | 0.000117 | 0.508857 | 0.274007354 | 0.686434129 |
| 15363-32 | Apo A-V | ENSG00000110243 | *APOA5* | 0.00012 | 0.508172 | 0.273152328 | 0.685945095 |
| 14006-36 | GNMT | ENSG00000124713 | *GNMT* | 0.000131 | 0.505764 | 0.270150248 | 0.684225091 |
| 3195-5 | Granulysin | ENSG00000115523 | *GNLY* | 0.000155 | 0.501039 | 0.26427428 | 0.680845144 |
| 8989-4 | SCUB1 | ENSG00000159307 | *SCUBE1* | 0.00016 | 0.500146 | 0.263165423 | 0.680205315 |
| 14624-51 | CTCF | ENSG00000102974 | *CTCF* | 0.000166 | -0.49913 | -0.679477233 | -0.261904954 |
| 12400-25 | UBE2T | ENSG00000077152 | *UBE2T* | 0.000171 | -0.49826 | -0.67885016 | -0.260820497 |
| 18882-7 | CSTN2 | ENSG00000158258 | *CLSTN2* | 0.000194 | 0.49459 | 0.256281105 | 0.676218684 |
| 15513-108 | Prostasin | ENSG00000052344 | *PRSS8* | 0.000195 | 0.494481 | 0.256146276 | 0.67614036 |
| 8304-50 | OPG | ENSG00000164761 | *TNFRSF11B* | 0.000226 | 0.49014 | 0.250783938 | 0.673017578 |
| 3028-36 | Ck-b-8-1 | ENSG00000274736 | *CCL23* | 0.000241 | 0.488218 | 0.24841488 | 0.671633135 |
| 3708-62 | a2-Macroglobulin | ENSG00000175899 | *A2M* | 0.000252 | 0.486902 | 0.246794701 | 0.670684622 |
| 15516-12 | SAA-4 | ENSG00000148965 | *SAA4* | 0.00027 | 0.484845 | 0.244263832 | 0.669200176 |
| 9370-69 | GGH | ENSG00000137563 | *GGH* | 0.000298 | 0.481893 | 0.240638902 | 0.667068101 |
| 15486-126 | ABP1 | ENSG00000002726 | *AOC1* | 0.0003 | 0.481707 | 0.240411008 | 0.666933826 |
| 13728-19 | ERP29 | ENSG00000089248 | *ERP29* | 0.000301 | -0.48154 | -0.666813554 | -0.24020692 |
| 5636-10 | MFAP4 | ENSG00000166482 | *MFAP4* | 0.000317 | 0.479959 | 0.238267179 | 0.665669333 |
| 2888-49 | C7 | ENSG00000112936 | *C7* | 0.000329 | 0.478882 | 0.236947684 | 0.664889834 |
| 11363-58 | AQP4 | ENSG00000171885 | *AQP4* | 0.000356 | -0.47645 | -0.663129908 | -0.233974368 |
| 2913-1 | MPIF-1 | ENSG00000274736 | *CCL23* | 0.000373 | 0.47508 | 0.232296637 | 0.662134749 |
| 17456-53 | GOLM1 | ENSG00000135052 | *GOLM1* | 0.000384 | 0.47417 | 0.231185621 | 0.661474906 |
| 13672-3 | HSP76 | ENSG00000173110 | *HSPA6* | 0.000415 | -0.47176 | -0.659727089 | -0.228248133 |
| 5742-14 | PPA6 | ENSG00000162836 | *ACP6* | 0.000431 | 0.470544 | 0.226763214 | 0.658841776 |
| 6227-1 | kallikrein 10 | ENSG00000129451 | *KLK10* | 0.000433 | 0.470421 | 0.226612392 | 0.658751789 |
| 5934-1 | Ferritin | ENSG00000167996 | *FTH1* | 0.00044 | -0.46991 | -0.658380542 | -0.22599039 |
| 5000-52 | LG3BP | ENSG00000108679 | *LGALS3BP* | 0.000474 | 0.467551 | 0.223119493 | 0.656664297 |
| 4481-34 | C4 | ENSG00000224389 | *C4B* | 0.000482 | 0.467028 | 0.22248392 | 0.656283738 |
| 12635-9 | TRDMT | ENSG00000107614 | *TRDMT1* | 0.000503 | -0.46569 | -0.655309564 | -0.220858626 |
| 13496-19 | HMCS1 | ENSG00000112972 | *HMGCS1* | 0.000508 | 0.465401 | 0.220506996 | 0.655098612 |
| 3184-25 | Coagulation Factor VII | ENSG00000057593 | *F7* | 0.000554 | 0.462632 | 0.217147444 | 0.653079713 |
| 5601-2 | PGRP-L | ENSG00000161031 | *PGLYRP2* | 0.000593 | 0.460443 | 0.214495647 | 0.651481746 |
| 19377-14 | NOE2 | ENSG00000105088 | *OLFM2* | 0.000685 | 0.455804 | 0.208887438 | 0.648089432 |
| 4971-1 | CATZ | ENSG00000101160 | *CTSZ* | 0.000689 | 0.455581 | 0.208617595 | 0.647925768 |
| 14249-68 | TRIM1 | ENSG00000080561 | *MID2* | 0.00074 | 0.453272 | 0.205832359 | 0.646234102 |
| 3607-71 | DKK3 | ENSG00000050165 | *DKK3* | 0.000843 | 0.448934 | 0.200611665 | 0.643051513 |
| 9191-8 | Trefoil factor 2 | ENSG00000160181 | *TFF2* | 0.000871 | 0.447859 | 0.199319131 | 0.642261204 |
| 19376-74 | NNMT | ENSG00000166741 | *NNMT* | 0.000974 | 0.444095 | 0.19480341 | 0.63949269 |
| 8975-26 | RCD1 | ENSG00000144580 | *CNOT9* | 0.001052 | 0.441473 | 0.191663617 | 0.637560913 |
| 12345-4 | APC10 | ENSG00000164162 | *ANAPC10* | 0.001076 | -0.4407 | -0.636994752 | -0.190745141 |
| 11109-56 | SVEP1 | ENSG00000165124 | *SVEP1* | 0.001133 | 0.438953 | 0.188651838 | 0.635702613 |
| 16914-104 | sCD14 | ENSG00000170458 | *CD14* | 0.001139 | 0.438772 | 0.188435511 | 0.635568937 |

| | | | | | | | |
|---|---|---|---|---|---|---|---|
| 6558-5 | COL10 | ENSG00000184374 | *COLEC10* | 0.001177 | 0.437636 | 0.187078251 | 0.634729628 |
| 11178-21 | SVEP1 | ENSG00000165124 | *SVEP1* | 0.001299 | 0.434204 | 0.182986636 | 0.632193027 |
| 11510-31 | Apo L1 | ENSG00000100342 | *APOL1* | 0.001313 | 0.43384 | 0.182553138 | 0.631923714 |
| 10666-7 | GNPTG | ENSG00000090581 | *GNPTG* | 0.001317 | 0.433724 | 0.182414517 | 0.631837572 |
| 17341-89 | THIC | ENSG00000120437 | *ACAT2* | 0.00132 | 0.433644 | 0.182319682 | 0.631778634 |
| 14133-93 | IL-1 sRII | ENSG00000115590 | *IL1R2* | 0.00146 | 0.430126 | 0.178135076 | 0.629172768 |
| 2692-74 | NPS-PLA2 | ENSG00000188257 | *PLA2G2A* | 0.001488 | 0.429439 | 0.177319297 | 0.628663576 |
| 6049-64 | PTPRS | ENSG00000105426 | *PTPRS* | 0.001568 | 0.427599 | 0.175135379 | 0.627298516 |
| 7854-38 | NR3L1 | ENSG00000188211 | *NCR3LG1* | 0.001579 | -0.42734 | -0.627104546 | -0.174825413 |
| 5018-68 | Peroxiredoxin-6 | ENSG00000117592 | *PRDX6* | 0.001655 | -0.42568 | -0.625870543 | -0.172855561 |
| 9829-91 | SULT 2A1 | ENSG00000105398 | *SULT2A1* | 0.001712 | 0.424449 | 0.17140306 | 0.624959176 |
| 16892-23 | ENPP2 | ENSG00000136960 | *ENPP2* | 0.001815 | 0.422362 | 0.168932842 | 0.623406403 |
| 19491-11 | DHB8 | ENSG00000204228 | *HSD17B8* | 0.002019 | -0.41848 | -0.620514421 | -0.164347324 |
| 13998-26 | PURA1 | ENSG00000185100 | *ADSS1* | 0.002046 | 0.418005 | 0.163788373 | 0.620161052 |
| 3283-21 | BGH3 | ENSG00000120708 | *TGFBI* | 0.002131 | 0.416502 | 0.162017019 | 0.61903998 |
| 12695-62 | KLH12 | ENSG00000117153 | *KLHL12* | 0.00221 | -0.41517 | -0.618043905 | -0.160445636 |
| 14747-9 | CRLF1 | ENSG00000006016 | *CRLF1* | 0.002288 | 0.413879 | 0.158929476 | 0.617081442 |
| 6480-1 | CANT1 | ENSG00000171302 | *CANT1* | 0.002334 | -0.41315 | -0.616534086 | -0.158068196 |
| 3326-58 | Nectin-like protein 2 | ENSG00000182985 | *CADM1* | 0.002385 | 0.412338 | 0.1571173 | 0.615929264 |
| 10558-26 | PCDH9 | ENSG00000184226 | *PCDH9* | 0.002525 | 0.410206 | 0.154613801 | 0.61433431 |
| 15391-114 | GAS-6 | ENSG00000183087 | *GAS6* | 0.002711 | 0.407541 | 0.151489036 | 0.612338268 |
| 9335-28 | PSG9 | ENSG00000183668 | *PSG9* | 0.002779 | 0.406597 | 0.150383216 | 0.611630481 |
| 3212-3 | ASAH2 | ENSG00000188611 | *ASAH2* | 0.002804 | 0.406266 | 0.149996051 | 0.611382499 |
| 10704-91 | LRMP | ENSG00000118308 | *IRAG2* | 0.002887 | -0.40516 | -0.610549602 | -0.148696716 |
| 8099-42 | SPON2 | ENSG00000159674 | *SPON2* | 0.00289 | 0.405117 | 0.148651209 | 0.610520412 |
| 6086-15 | CRDL2 | ENSG00000054938 | *CHRDL2* | 0.003002 | 0.40367 | 0.146958428 | 0.609433728 |
| 15548-35 | SERA | ENSG00000092621 | *PHGDH* | 0.003393 | -0.39896 | -0.605892006 | -0.141460053 |
| 17460-51 | Mx1 | ENSG00000157601 | *MX1* | 0.003458 | 0.398216 | 0.14059425 | 0.605332625 |
| 5649-83 | PSG4 | ENSG00000243137 | *PSG4* | 0.003504 | 0.397705 | 0.139999413 | 0.604948044 |
| 4763-31 | Afamin | ENSG00000079557 | *AFM* | 0.003535 | 0.397359 | 0.139596442 | 0.604687388 |
| 15556-49 | Alpha-amylase 2B | ENSG00000240038 | *AMY2B* | 0.00361 | -0.39654 | -0.604073644 | -0.138648212 |
| 3070-1 | IL-2 | ENSG00000109471 | *IL2* | 0.003619 | 0.396444 | 0.13853176 | 0.603998232 |
| 9313-27 | CBLN1 | ENSG00000102924 | *CBLN1* | 0.003728 | 0.395283 | 0.137180959 | 0.603122873 |
| 18380-78 | Albumin | ENSG00000163631 | *ALB* | 0.003758 | 0.39497 | 0.136817263 | 0.602886995 |
| 9002-36 | SPA11 | ENSG00000186910 | *SERPINA11* | 0.00376 | 0.394951 | 0.13679481 | 0.602872431 |
| 4374-45 | MIC-1 | ENSG00000130513 | *GDF15* | 0.003823 | 0.394296 | 0.136032968 | 0.602378057 |
| 19553-14 | STX1a | ENSG00000106089 | *STX1A* | 0.003839 | -0.39414 | -0.602258495 | -0.135848803 |
| 8843-34 | MRF | ENSG00000124920 | *MYRF* | 0.003861 | -0.39391 | -0.60208588 | -0.135582974 |
| 3580-25 | a1-Antitrypsin | ENSG00000197249 | *SERPINA1* | 0.003942 | 0.393097 | 0.134640604 | 0.601473601 |
| 13463-1 | PXDN | ENSG00000130508 | *PXDN* | 0.004136 | 0.39119 | 0.132426985 | 0.600033204 |
| 15585-304 | fibulin 5 | ENSG00000140092 | *FBLN5* | 0.004299 | 0.389656 | 0.130648527 | 0.598873765 |
| 7211-2 | RNase 1 | ENSG00000129538 | *RNASE1* | 0.004327 | 0.389397 | 0.130348686 | 0.598678094 |
| 2994-71 | IL-1Rrp2 | ENSG00000115598 | *IL1RL2* | 0.004394 | 0.388781 | 0.129634335 | 0.598211697 |
| 3447-64 | IL-8 | ENSG00000169429 | *CXCL8* | 0.004414 | 0.388606 | 0.129431752 | 0.598079374 |
| 4153-11 | alpha-1-antichymotrypsin complex | ENSG00000196136 | *SERPINA3* | 0.004549 | 0.387396 | 0.12803175 | 0.597164219 |
| 15515-2 | SAA | ENSG00000173432 | *SAA1* | 0.004633 | 0.386662 | 0.127182025 | 0.596608174 |

| | | | | | | | |
|---|---|---|---|---|---|---|---|
| 8956-96 | SREC-II | ENSG00000244486 | *SCARF2* | 0.00481 | 0.385149 | 0.125432875 | 0.595462141 |
| 5752-63 | SUSD3 | ENSG00000157303 | *SUSD3* | 0.0049 | 0.384398 | 0.124564553 | 0.594892509 |
| 14132-21 | HHLA2 | ENSG00000114455 | *HHLA2* | 0.004975 | 0.38378 | 0.123850586 | 0.594423783 |
| 19282-3 | RAB13 | ENSG00000143545 | *RAB13* | 0.005047 | -0.3832 | -0.593982313 | -0.123178582 |
| 6410-26 | INSL4 | ENSG00000120211 | *INSL4* | 0.005108 | 0.382708 | 0.122614098 | 0.593611259 |
| 9876-2 | aldolase C | ENSG00000109107 | *ALDOC* | 0.005146 | 0.382406 | 0.122264982 | 0.593381673 |
| 18832-65 | SAA2 | ENSG00000134339 | *SAA2* | 0.005307 | 0.381153 | 0.120820334 | 0.592430827 |
| 2950-57 | IGFBP-4 | ENSG00000141753 | *IGFBP4* | 0.005354 | 0.380787 | 0.120397688 | 0.592152399 |
| 8589-13 | CDCP1 | ENSG00000163814 | *CDCP1* | 0.005356 | 0.380773 | 0.120381661 | 0.592141838 |
| 13427-66 | MA1C1 | ENSG00000117643 | *MAN1C1* | 0.005472 | 0.379894 | 0.119368825 | 0.59147414 |
| 3481-87 | XPNPEP1 | ENSG00000108039 | *XPNPEP1* | 0.005723 | 0.378047 | 0.117242319 | 0.59007016 |
| 13631-1 | RB binding protein 5 | ENSG00000117222 | *RBBP5* | 0.005765 | -0.37775 | -0.589843204 | -0.116898972 |
| 8958-51 | CHL1 | ENSG00000134121 | *CHL1* | 0.005808 | 0.377442 | 0.116546547 | 0.58961017 |
| 18435-4 | UBX2B | ENSG00000215114 | *UBXN2B* | 0.005893 | -0.37683 | -0.589148121 | -0.115848129 |
| 11406-82 | ACAD8 | ENSG00000151498 | *ACAD8* | 0.00609 | 0.37547 | 0.114279026 | 0.588108928 |
| 11167-6 | MTMR1 | ENSG00000063601 | *MTMR1* | 0.006306 | -0.37402 | -0.587001965 | -0.112610192 |
| 12956-4 | KBP | ENSG00000198954 | *KIFBP* | 0.006323 | 0.373907 | 0.112484564 | 0.586918562 |
| 15310-61 | PRC1 | ENSG00000198901 | *PRC1* | 0.006387 | 0.373484 | 0.111998781 | 0.586595963 |
| 17748-21 | QORX | ENSG00000115129 | *TP53I3* | 0.006437 | 0.373157 | 0.111623513 | 0.58634665 |
| 10890-135 | LCTL | ENSG00000188501 | *LCTL* | 0.006461 | 0.373003 | 0.111447293 | 0.586229547 |
| 12815-9 | SYIC | ENSG00000196305 | *IARS1* | 0.006581 | -0.37223 | -0.585641792 | -0.110563281 |
| 19617-5 | LTB4DH | ENSG00000106853 | *PTGR1* | 0.006583 | 0.372218 | 0.110547069 | 0.585631008 |
| 9986-14 | Neuropeptide W | ENSG00000183971 | *NPW* | 0.006653 | 0.371771 | 0.110034732 | 0.585290136 |
| 3143-3 | sCD4 | ENSG00000010610 | *CD4* | 0.006701 | 0.371467 | 0.109685786 | 0.585057875 |
| 5092-51 | JAG1 | ENSG00000101384 | *JAG1* | 0.006769 | 0.371044 | 0.109201304 | 0.584735273 |
| 18876-77 | CHST4 | ENSG00000140835 | *CHST4* | 0.006792 | 0.370899 | 0.109035231 | 0.584624655 |
| 8252-2 | NOTUM | ENSG00000185269 | *NOTUM* | 0.006805 | 0.370822 | 0.108946712 | 0.584565686 |
| 5029-3 | SEPR | ENSG00000078098 | *FAP* | 0.006905 | 0.370202 | 0.108237006 | 0.584092726 |
| 11667-29 | TENC1 | ENSG00000111077 | *TNS2* | 0.007046 | 0.369349 | 0.107259688 | 0.583440895 |
| 8245-27 | sICAM-5 | ENSG00000105376 | *ICAM5* | 0.0071 | 0.369023 | 0.106886569 | 0.583191877 |
| 18880-81 | Collagen Type III | ENSG00000168542 | *COL3A1* | 0.007134 | 0.368822 | 0.106656522 | 0.583038301 |
| 13098-93 | VEGF-D | ENSG00000165197 | *VEGFD* | 0.00728 | 0.367956 | 0.105666058 | 0.58237669 |
| 16616-137 | ENOB | ENSG00000108515 | *ENO3* | 0.00744 | 0.367034 | 0.104611798 | 0.581671771 |
| 9758-17 | RS4X | ENSG00000198034 | *RPS4X* | 0.007541 | -0.36646 | -0.581230085 | -0.103951769 |
| 18202-22 | CD37 | ENSG00000104894 | *CD37* | 0.007628 | -0.36596 | -0.58085396 | -0.103390042 |
| 15606-19 | Keratin 19 | ENSG00000171345 | *KRT19* | 0.007704 | 0.365538 | 0.102902973 | 0.58052766 |
| 10037-98 | SIG12 | ENSG00000254521 | *SIGLEC12* | 0.007711 | 0.365502 | 0.102861978 | 0.580500189 |
| 6039-24 | CRHBP | ENSG00000145708 | *CRHBP* | 0.007932 | 0.364285 | 0.101472237 | 0.579568285 |
| 3000-66 | MBL | ENSG00000165471 | *MBL2* | 0.008038 | 0.363711 | 0.100817601 | 0.579128879 |
| 14151-4 | UCRP | ENSG00000187608 | *ISG15* | 0.008656 | 0.3605 | 0.097158515 | 0.576667691 |
| 3890-8 | LDH-H 1 | ENSG00000111716 | *LDHB* | 0.008791 | -0.35983 | -0.576152409 | -0.096394074 |
| 8459-1 | BMP-6 | ENSG00000153162 | *BMP6* | 0.008794 | -0.35981 | -0.576139183 | -0.096374461 |
| 2190-55 | Coagulation Factor XI | ENSG00000088926 | *F11* | 0.008818 | 0.359696 | 0.096242483 | 0.576050182 |
| 4557-61 | KIRR3 | ENSG00000149571 | *KIRREL3* | 0.008821 | 0.35968 | 0.096224916 | 0.576038334 |
| 4276-1 | prostatic binding protein | ENSG00000089220 | *PEBP1* | 0.008944 | -0.35907 | -0.575572704 | -0.095534736 |
| 9583-17 | RNF24 | ENSG00000101236 | *RNF24* | 0.008949 | 0.359051 | 0.095509317 | 0.575555549 |

| | | | | | | | |
|---|---|---|---|---|---|---|---|
| 17835-28 | ANX13 | ENSG00000104537 | *ANXA13* | 0.009028 | -0.35866 | -0.575256528 | -0.095066347 |
| 11454-87 | EIF3G | ENSG00000130811 | *EIF3G* | 0.009231 | -0.35769 | -0.57450809 | -0.093958445 |
| 6367-66 | fibromodulin | ENSG00000122176 | *FMOD* | 0.009231 | 0.357687 | 0.09395768 | 0.574507574 |
| 11372-2 | ZNF18 | ENSG00000154957 | *ZNF18* | 0.009256 | -0.35757 | -0.574418404 | -0.093825762 |
| 10514-5 | PGD2 synthase | ENSG00000107317 | *PTGDS* | 0.009417 | 0.356808 | 0.09295992 | 0.573832853 |
| 14051-54 | FOXC2 | ENSG00000176692 | *FOXC2* | 0.009599 | 0.355966 | 0.09200331 | 0.573185347 |
| 9837-6 | NAD(P)H dehydrogenase | ENSG00000181019 | *NQO1* | 0.009637 | 0.355792 | 0.091805691 | 0.573051508 |
| 7928-183 | TPST1 | ENSG00000169902 | *TPST1* | 0.009664 | 0.355667 | 0.091664184 | 0.572955656 |
| 13961-18 | KIF3A | ENSG00000131437 | *KIF3A* | 0.009778 | 0.355148 | 0.091075141 | 0.572556516 |
| 14107-1 | MTHFS | ENSG00000136371 | *MTHFS* | 0.009814 | -0.35499 | -0.572432914 | -0.0908928 |
| 9125-23 | MASP3 | ENSG00000127241 | *MASP1* | 0.009927 | 0.35448 | 0.090316473 | 0.5720421 |
| 11383-41 | Keratin 7 | ENSG00000135480 | *KRT7* | 0.009988 | 0.354206 | 0.090006022 | 0.571831489 |

**Supplementary Table S5. Proteo-transcriptomics signature and differential gene expression in cohort of 206 NAFLD patients (Govaere et al 2020; Benjamini-Hochberg false discovery rate)**

| | | | | | | F3-4 vs F0-2 (RNAseq n=206) | | | NAS4>=4 vs <4 (RNAseq n=206) | | |
|---|---|---|---|---|---|---|---|---|---|---|---|
| **Circulating Proteome** | **Soma ID** | **Protein ID** | **Ens ID** | **Gene ID** | **Name** | **log2FC** | **p-value** | **adj p-value** | **log2FC** | **p-value** | **adj p-value** |
| F34 and NAS4 | 3339-33 | TSP2 | ENSG00000186340 | *THBS2* | thrombospondin 2 | 1.15 | 3.05E-16 | 8.23E-13 | 0.88 | 4.43E-08 | 6.55E-05 |
| F34 and NAS4 | 12370-30 | Apo F | ENSG00000175336 | *APOF* | apolipoprotein F | -0.25 | 2.60E-03 | 2.62E-02 | -0.38 | 1.46E-05 | 2.24E-03 |
| F34 and NAS4 | 6379-62 | ATL2 | ENSG00000197859 | *ADAMTSL2* | ADAMTS-like 2 | 0.54 | 3.82E-08 | 3.57E-06 | 0.28 | 8.14E-03 | 9.67E-02 |
| F34 and NAS4 | 6471-53 | FHR4 | ENSG00000134365 | *CFHR4* | complement factor H-related 4 | -0.42 | 6.66E-04 | 9.58E-03 | -0.27 | 4.08E-02 | 2.31E-01 |
| F34 and NAS4 | 16300-4 | TREM2 | ENSG00000095970 | *TREM2* | triggering receptor expressed on myeloid cells 2 | 0.83 | 5.59E-04 | 8.42E-03 | 1.59 | 7.71E-11 | 8.30E-07 |
| F34 and NAS4 | 16081-38 | Aldose reductase-like | ENSG00000198074 | *AKR1B10* | aldo-keto reductase family 1, member B10 (aldose reductase) | 1.88 | 9.94E-08 | 7.78E-06 | 1.95 | 2.94E-07 | 2.21E-04 |
| F34 and NAS4 | 9829-91 | SULT 2A1 | ENSG00000105398 | *SULT2A1* | sulfotransferase family, cytosolic, 2A, DHEA-preferring, member 1 | 0.14 | 1.25E-01 | 3.63E-01 | -0.01 | 9.48E-01 | 9.84E-01 |
| F34 and NAS4 | 19617-5 | LTB4DH | ENSG00000106853 | *PTGR1* | prostaglandin reductase 1 | -0.03 | 5.26E-01 | 7.67E-01 | 0.06 | 2.88E-01 | 6.17E-01 |
| NAS4 only | 13998-26 | PURA1 | ENSG00000185100 | *ADSSL1* | adenylosuccinate synthase like 1 | -0.23 | 4.72E-03 | 4.10E-02 | 0.00 | 9.92E-01 | 9.97E-01 |
| NAS4 only | 16616-137 | ENOB | ENSG00000108515 | *ENO3* | enolase 3 (beta, muscle) | -0.17 | 6.53E-02 | 2.45E-01 | 0.03 | 7.51E-01 | 9.09E-01 |
| F34 only | 4929-55 | SHBG | ENSG00000129214 | *SHBG* | sex hormone-binding globulin | 0.35 | 1.97E-02 | 1.12E-01 | -0.26 | 1.03E-01 | 3.74E-01 |
| F34 only | 6580-29 | PZP | ENSG00000126838 | *PZP* | pregnancy-zone protein | -1.04 | 7.31E-04 | 1.02E-02 | -0.87 | 7.22E-03 | 9.02E-02 |
| F34 only | 4435-66 | ENPP7 | ENSG00000182156 | *ENPP7* | ectonucleotide pyrophosphatase/phosphodiesterase 7 | 0.40 | 2.38E-03 | 2.46E-02 | 0.18 | 2.10E-01 | 5.32E-01 |
| F34 only | 11646-4 | CHST9 | ENSG00000154080 | *CHST9* | carbohydrate (N-acetylgalactosamine 4-0) sulfotransferase 9 | 0.89 | 4.48E-10 | 8.88E-08 | 0.77 | 9.77E-07 | 4.83E-04 |
| F34 only | 6605-17 | IGFALS | ENSG00000099769 | *IGFALS* | insulin-like growth factor binding protein, acid labile subunit | -0.65 | 1.82E-06 | 8.58E-05 | -0.48 | 8.87E-04 | 2.59E-02 |
| F34 only | 3320-49 | IGFBP-7 | ENSG00000163453 | *IGFBP7* | insulin-like growth factor binding protein 7 | 0.62 | 2.26E-11 | 7.86E-09 | 0.42 | 3.18E-05 | 3.53E-03 |
| F34 only | 13717-15 | FCN2 | ENSG00000160339 | *FCN2* | ficolin (collagen/fibrinogen domain containing lectin) 2 | -0.64 | 1.43E-12 | 6.34E-10 | -0.20 | 5.07E-02 | 2.60E-01 |
| F34 only | 7179-69 | NFASC | ENSG00000163531 | *NFASC* | neurofascin | 0.89 | 3.04E-17 | 1.97E-13 | 0.33 | 5.88E-03 | 8.02E-02 |
| F34 only | 18893-26 | GPR56 | ENSG00000205336 | *ADGRG1* | adhesion G protein-coupled receptor G1 | 0.87 | 4.54E-09 | 6.24E-07 | 0.47 | 4.98E-03 | 7.28E-02 |
| F34 only | 11104-13 | YKL-40 | ENSG00000133048 | *CHI3L1* | chitinase 3-like 1 (cartilage glycoprotein-39) | 1.07 | 5.56E-05 | 1.37E-03 | 0.90 | 1.85E-03 | 4.04E-02 |
| F34 only | 15363-32 | Apo A-V | ENSG00000110243 | *APOA5* | apolipoprotein A-V | -0.32 | 9.09E-05 | 2.01E-03 | -0.22 | 1.09E-02 | 1.14E-01 |
| F34 only | 18882-7 | CSTN2 | ENSG00000158258 | *CLSTN2* | calsyntenin 2 | 0.84 | 2.74E-07 | 1.81E-05 | 0.53 | 3.00E-03 | 5.41E-02 |
| F34 only | 8304-5 | OPG | ENSG00000164761 | *TNFRSF11B* | tumor necrosis factor receptor superfamily, member 11b | 0.26 | 6.13E-03 | 4.95E-02 | 0.23 | 2.43E-02 | 1.75E-01 |
| F34 only | 5636-10 | MFAP4 | ENSG00000166482 | *MFAP4* | microfibrillar-associated protein 4 | 0.77 | 2.46E-10 | 5.40E-08 | 0.60 | 6.16E-06 | 1.41E-03 |
| F34 only | 17456-53 | GOLM1 | ENSG00000135052 | *GOLM1* | golgi membrane protein 1 | 0.68 | 2.44E-06 | 1.10E-04 | 0.54 | 5.39E-04 | 1.91E-02 |
| F34 only | 5000-52 | LG3BP | ENSG00000108679 | *LGALS3BP* | lectin, galactoside-binding, soluble, 3 binding protein | 0.19 | 3.36E-02 | 1.61E-01 | 0.16 | 9.68E-02 | 3.63E-01 |
| F34 only | 11178-21 | SVEP1 | ENSG00000165124 | *SVEP1* | sushi, von Willebrand factor type A, EGF and pentraxin domain containing 1 | 0.55 | 2.09E-09 | 3.33E-07 | 0.37 | 2.92E-04 | 1.32E-02 |
| F34 only | 15556-49 | Alpha-amylase 2B | ENSG00000240038 | *AMY2B* | amylase, alpha 2B (pancreatic) | 0.03 | 6.14E-01 | 8.22E-01 | -0.11 | 1.26E-01 | 4.17E-01 |
| F34 only | 4374-45 | MIC-1 | ENSG00000130513 | *GDF15* | growth differentiation factor 15 | 0.79 | 3.77E-09 | 5.28E-07 | 0.58 | 6.76E-05 | 5.41E-03 |
| F34 only | 3447-64 | IL-8 | ENSG00000169429 | *CXCL8* | chemokine (C-X-C motif) ligand 8 | 1.57 | 2.86E-10 | 6.07E-08 | 1.00 | 2.89E-04 | 1.32E-02 |
| F34 only | 9986-14 | Neuropeptide W | ENSG00000183971 | *NPW* | neuropeptide W | 0.73 | 2.64E-04 | 4.67E-03 | 0.23 | 2.81E-01 | 6.10E-01 |

**Supplementary Table S6. Performance of the proteome classification model in comparison with other scores**

***cut-off >-0.4491 (Sensitivity 0.84 and 1-Specificity 0.14 in Discovery Cohort, Sensitivity 0.91 and 1-Specificity 0.49 in Validation Cohort)***

| **Discovery_cohort (n=191)** | | | | |
|---|---|---|---|---|
| Classification model | Rule-in | positive predictive value | 74/97 | 0.76 |
| | Rule-out | negative predictive value | 14/94 | 0.85 |
| FIB-4 (>1.3) | Rule-in | positive predictive value | 74/97 | 0.59 |
| | Rule-out | negative predictive value | 14/94 | 0.68 |

| **Subset_Discovery_cohort (n=35)** | | | | |
|---|---|---|---|---|
| Classification model | Rule-in | positive predictive value | 12/15 | 0.80 |
| | Rule-out | negative predictive value | 2/20 | 0.90 |
| FAST | Rule-in (>0.67) | positive predictive value | 12/18 | 0.67 |
| | Rule-out (<=0.35) | negative predictive value | 2/17 | 0.88 |

| **Validation_cohort (n=115)** | | | | |
|---|---|---|---|---|
| Classification model | Rule-in | positive predictive value | 39/74 | 0.53 |
| | Rule-out | negative predictive value | 4/41 | 0.90 |
| FIB-4 (>1.3) | Rule-in | positive predictive value | 25/54 | 0.46 |
| | Rule-out | negative predictive value | 18/61 | 0.70 |

| **Subset_Validation_cohort (n=15)** | | | | |
|---|---|---|---|---|
| Classification model | Rule-in | positive predictive value | 5/9 | 0.56 |
| | Rule-out | negative predictive value | 4/4 | 1.00 |
| FAST | Rule-in (>0.67) | positive predictive value | 5/8 | 0.63 |
| | Rule-out (<=0.35) | negative predictive value | 5/5 | 1.00 |

| The LITMUS Investigators / Group Authors | |
|---|---|
| Newcastle University | Quentin M. Anstee<br>Ann K. Daly<br>Olivier Govaere<br>Simon Cockell<br>Dina Tiniakos †<br>Pierre Bedossa †<br>Alastair Burt †<br>Fiona Oakley<br>Heather J. Cordell<br>Christopher P. Day<br>Kristy Wonders<br>Paolo Missier<br>Matthew McTeer<br>Luke Vale<br>Yemi Oluboyede<br>Matt Breckons |
| AMC Amsterdam | Patrick M. Bossuyt<br>Hadi Zafarmand<br>Yasaman Vali<br>Jenny Lee<br>Max Nieuwdorp<br>Adriaan G. Holleboom<br>Joanne Verheij † |
| Institute of Cardiometabolism And Nutrition | Vlad Ratziu<br>Karine Clément<br>Rafael Patino-Navarrete<br>Raluca Pais |
| Hôpital Beaujon, Assistance Publique Hopitaux de Paris | Valerie Paradis † |
| University Medical Center Mainz | Detlef Schuppan<br>Jörn M. Schattenberg<br>Rambabu Surabattula<br>Sudha Myneni<br>Beate K. Straub † |
| University of Cambridge | Toni Vidal-Puig<br>Michele Vacca<br>Sergio Rodrigues-Cuenca<br>Mike Allison<br>Ioannis Kamzolas<br>Evangelia Petsalaki<br>Mark Campbell<br>Chris J. Lelliott<br>Susan Davies † |
| Örebro University | Matej Orešič<br>Tuulia Hyötyläinen<br>Aiden McGlinchey |
| Center for Cooperative Research in Biosciences | Jose M. Mato<br>Óscar Millet |
| University of Bern | Jean-François Dufour<br>Annalisa Berzigotti<br>Mojgan Masoodi |
| University of Oxford | Michael Pavlides<br>Stephen Harrison<br>Stefan Neubauer<br>Jeremy Cobbold<br>Ferenc Mozes<br>Salma Akhtar<br>Seliat Olodo-Atitebi |
| Perspectum | Rajarshi Banerjee<br>Matt Kelly<br>Elizabeth Shumbayawonda<br>Andrea Dennis<br>Anneli Andersson<br>Ioan Wigley |

| | |
|---|---|
| Servicio Andaluz de Salud, Seville | Manuel Romero-Gómez<br>Emilio Gómez-González<br>Javier Ampuero<br>Javier Castell<br>Rocío Gallego-Durán<br>Isabel Fernández<br>Rocío Montero-Vallejo |
| Nordic Bioscience | Morten Karsdal<br>Daniel Guldager Kring Rasmussen<br>Diana Julie Leeming<br>Antonia Sinisi<br>Kishwar Musa |
| Integrated Biobank of Luxembourg | Estelle Sandt<br>Manuela Tonini |
| University of Torino | Elisabetta Bugianesi<br>Chiara Rosso<br>Angelo Armandi |
| Università degli Studi di Firenze | Fabio Marra |
| Consiglio Nazionale delle Ricerche | Amalia Gastaldelli |
| Università Politecnica delle Marche | Gianluca Svegliati |
| University Hospital of Angers | Jérôme Boursier |
| Antwerp University Hospital | Sven Francque<br>Luisa Vonghia<br>Ann Driessen † |
| Linköping University | Mattias Ekstedt<br>Stergios Kechagias |
| University of Helsinki | Hannele Yki-Järvinen<br>Kimmo Porthan<br>Johanna Arola † |
| UMC Utrecht | Saskia van Mil |
| Medical School of National & Kapodistrian University of Athens | George Papatheodoridis |
| Faculdade de Medicina, Universidade de Lisboa | Helena Cortez-Pinto |
| Faculty of Pharmacy, Universidade de Lisboa | Cecilia M. P. Rodrigues |
| Università degli Studi di Milano | Luca Valenti<br>Serena Pelusi |
| Università degli Studi di Palermo | Salvatore Petta<br>Grazia Pennisi |
| Università Cattolica del Sacro Cuore | Luca Miele |
| University Hospital Würzburg | Andreas Geier |
| RWTH Aachen University Hospital | Christian Trautwein<br>Johanna Reißing |
| University of Nottingham | Guruprasad P. Aithal<br>Susan Francis<br>Naaventhan Palaniyappan<br>Christopher Bradley |
| Antaros Medical | Paul Hockings<br>Moritz Schneider |
| University Hospitals Birmingham NHS Foundation Trust | Philip Newsome<br>Stefan Hübscher † |
| iXscient | David Wenn |
| Genfit | Christian Rosenquist |
| Intercept Pharma | Aldo Trylesinski |

| | |
|---|---|
| OWL | Rebeca Mayo<br>Cristina Alonso |
| Eli Lilly and Company | Kevin Duffin<br>James W. Perfield<br>Yu Chen |
| Pfizer | Carla Yunis<br>Theresa Tuthill<br>Magdalena Alicia Harrington<br>Melissa Miller<br>Yan Chen<br>Euan James McLeod<br>Trenton Ross<br>Barbara Bernardo |
| Boehringer-Ingelheim | Corinna Schölch<br>Judith Ertle<br>Ramy Younes<br>Anouk Oldenburger<br>Harvey Coxson |
| Somalogic | Rachel Ostroff<br>Leigh Alexander<br>Hannah Biegel |
| Novo Nordisk | Mette Skalshøi Kjær<br>Lea Mørch Harder<br>Peter Davidsen |
| Ellegaard Göttingen Minipigs | Jens Ellegaard |
| Novartis Pharma AG | Maria-Magdalena Balp<br>Clifford Brass<br>Lori Jennings<br>Miljen Martic<br>Jürgen Löffler<br>Douglas Applegate |
| AstraZeneca | Sudha Shankar<br>Richard Torstenson<br>Daniel Lindén |
| Echosens | Céline Fournier-Poizat<br>Anne Llorca |
| Resoundant | Michael Kalutkiewicz<br>Kay Pepin<br>Richard Ehman |
| Bristol-Myers Squibb | Gerald Horan |
| HistoIndex | Gideon Ho<br>Dean Tai<br>Elaine Chng |
| Gilead | Scott D. Patterson<br>Andrew Billin |
| RTI-HS | Lynda Doward<br>James Twiss |
| Takeda Pharmaceuticals Company Ltd. | Paresh Thakker<br>Zoltan Derdak |
| AbbVie | Henrik Landgren |
| Medical University of Graz | Carolin Lackner † |
| University of Groningen | Annette Gouw † |
| Aristotle University of Thessaloniki | Prodromos Hytiroglou † |

† Member of the LITMUS Histopathology Group (LHG)
