# Supplementary material for: A proteo-transcriptomic map of non-alcoholic fatty liver disease signatures
Source: Nat Metab. 2023 Apr 10;5(4):572–8. doi: 10.1038/s42255-023-00775-1 (PMC10132975; doi:10.1038/s42255-023-00775-1)
Supplement: Supplementary file 2 — Reporting Summary [file 42255_2023_775_MOESM2_ESM.pdf]

nature research

Corresponding author(s): Olivier Govaere, Quentin Anstee

Last updated by author(s): Feb 9, 2023

Reporting Summary

Nature Research wishes to improve the reproducibility of the work that we publish. This form provides structure for consistency and transparency in reporting. For further information on Nature Research policies, see our Editorial Policies and the Editorial Policy Checklist.

Statistics

For all statistical analyses, confirm that the following items are present in the figure legend, table legend, main text, or Methods section.

n/a	Confirmed	
☐	☒	The exact sample size (n) for each experimental group/condition, given as a discrete number and unit of measurement
☐	☒	A statement on whether measurements were taken from distinct samples or whether the same sample was measured repeatedly
☐	☒	The statistical test(s) used AND whether they are one- or two-sided<br>*Only common tests should be described solely by name; describe more complex techniques in the Methods section.*
☐	☒	A description of all covariates tested
☐	☒	A description of any assumptions or corrections, such as tests of normality and adjustment for multiple comparisons
☐	☒	A full description of the statistical parameters including central tendency (e.g. means) or other basic estimates (e.g. regression coefficient) AND variation (e.g. standard deviation) or associated estimates of uncertainty (e.g. confidence intervals)
☐	☒	For null hypothesis testing, the test statistic (e.g. F, t, r) with confidence intervals, effect sizes, degrees of freedom and P value noted<br>*Give P values as exact values whenever suitable.*
☒	☐	For Bayesian analysis, information on the choice of priors and Markov chain Monte Carlo settings
☐	☒	For hierarchical and complex designs, identification of the appropriate level for tests and full reporting of outcomes
☒	☐	Estimates of effect sizes (e.g. Cohen's d, Pearson's r), indicating how they were calculated

Our web collection on statistics for biologists contains articles on many of the points above.

Software and code

Policy information about availability of computer code

Data collection	SomaScan platform and Illumina NextSeq 550 System
Data analysis	R, Graphpad and IBM SPSS vs26

For manuscripts utilizing custom algorithms or software that are central to the research but not yet described in published literature, software must be made available to editors and reviewers. We strongly encourage code deposition in a community repository (e.g. GitHub). See the Nature Research guidelines for submitting code & software for further information.

Data

Policy information about availability of data

All manuscripts must include a data availability statement. This statement should provide the following information, where applicable:

- Accession codes, unique identifiers, or web links for publicly available datasets
- A list of figures that have associated raw data
- A description of any restrictions on data availability

SomaLogic data and images of the immunohistochemistry have been provided as Data Source Files alongside the paper. Availability of RNAseq data has been stated in the manuscript with the NCBI GEO repository number.

Field-specific reporting

Please select the one below that is the best fit for your research. If you are not sure, read the appropriate sections before making your selection.

☒ Life sciences ☐ Behavioural & social sciences ☐ Ecological, evolutionary & environmental sciences

For a reference copy of the document with all sections, see nature.com/documents/nr-reporting-summary-flat.pdf

Life sciences study design

All studies must disclose on these points even when the disclosure is negative.

Sample size	191 plasma samples and 51 paired liver biopsies, 115 serum samples and 30 FFPE biopsies
Data exclusions	No samples excluded.
Replication	Not applicable.
Randomization	Not random.
Blinding	Histological scoring has been done blinded.

Reporting for specific materials, systems and methods

We require information from authors about some types of materials, experimental systems and methods used in many studies. Here, indicate whether each material, system or method listed is relevant to your study. If you are not sure if a list item applies to your research, read the appropriate section before selecting a response.

Materials & experimental systems

n/a	Involved in the study	
☐	☒	Antibodies
☒	☐	Eukaryotic cell lines
☒	☐	Palaeontology and archaeology
☒	☐	Animals and other organisms
☐	☒	Human research participants
☐	☒	Clinical data
☒	☐	Dual use research of concern

Methods

n/a	Involved in the study	
☒	☐	ChIP-seq
☒	☐	Flow cytometry
☒	☐	MRI-based neuroimaging

Antibodies

Antibodies used	Recombinant Anti-AKR1B10 antibody (ab232623)
Validation	https://www.abcam.com/akr1b10-antibody-epr14421-bsa-and-azide-free-ab232623.html

Human research participants

Policy information about studies involving human research participants

Population characteristics	336 histologically characterised NAFLD patient cases were derived from the European NAFLD Registry (NCT04442334).
Recruitment	Patients have been treated and diagnosed for NAFLD based on histology at specialised centres including Angers & Paris (France), Mainz (Germany), Turin (Italy), Linköping (Sweden) and Newcastle upon Tyne (UK).
Ethics oversight	This study has been approved by the relevant Ethical Committees in the participating centres and all patients having provided informed consent.

Note that full information on the approval of the study protocol must also be provided in the manuscript.

Clinical data

Policy information about clinical studies

All manuscripts should comply with the ICMJE guidelines for publication of clinical research and a completed CONSORT checklist must be included with all submissions.

Clinical trial registration	NCT04442334
Study protocol	Hardy, T., et al. The European NAFLD Registry: A real-world longitudinal cohort study of nonalcoholic fatty liver disease. Contemp Clin Trials 98, 106175 (2020).
Data collection	The cohort comprised 306 cases with plasma/serum samples taken less than three months from the time of biopsy. A subset, comprising 51 of these cases had frozen liver tissue available for RNA extraction. A cohort of 30 FFPE liver biopsies were used to validate protein targets on tissue level. All liver samples were centrally scored according to the semi-quantitative NASH-CRN Scoring System by an expert liver pathologist.<br>Sex and/or gender of participants was determined based on self-report.
Outcomes	No significant differences in sex/gender were observed when stratifying patients based upon T2DM, fibrosis or disease activity status/stage. Sex was included as a variable in the logistic modeling but was not significant.<br>The outcome was a non-invasive diagnostics tool to identify patients with at-risk NAFLD based on BMI, type 2 diabetes status, and four circulating proteins.
