# Supplementary material for: A proteo-transcriptomic map of non-alcoholic fatty liver disease signatures
Source: Nat Metab. 2023 Apr 10;5(4):572–8. doi: 10.1038/s42255-023-00775-1 (PMC10132975; doi:10.1038/s42255-023-00775-1)

Source data file:
Overview immunohistochemical staining for AKR1B10 in 30 biopsies from patients with NAFLD.

Patient 1 – NASH F4

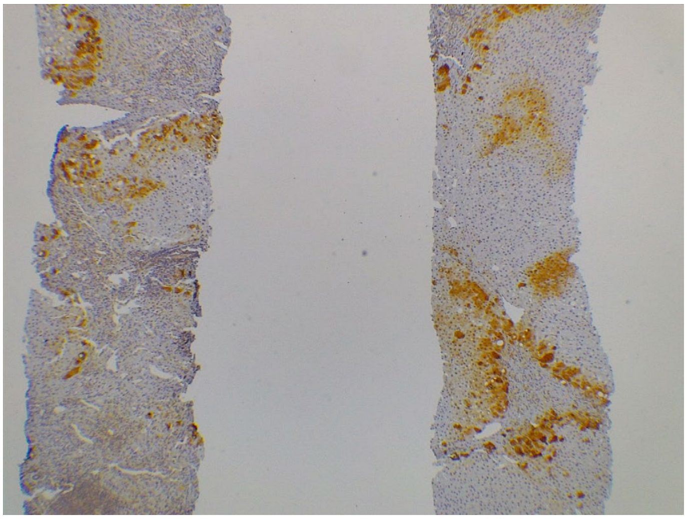

Patient 2 – NASH F4

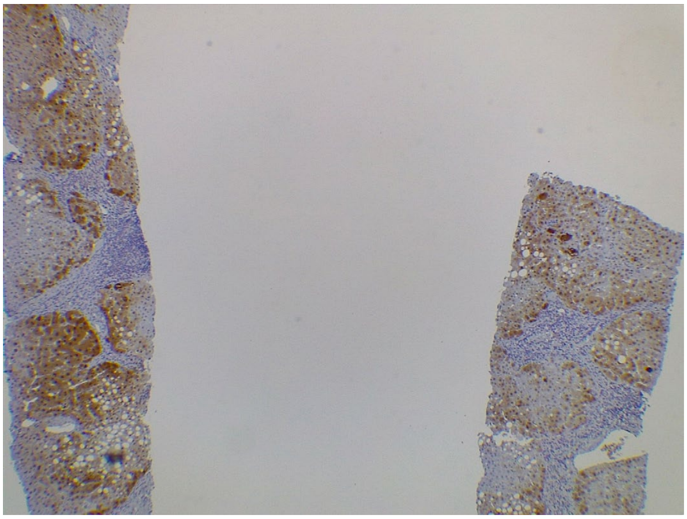

Patient 3 – NASH F4

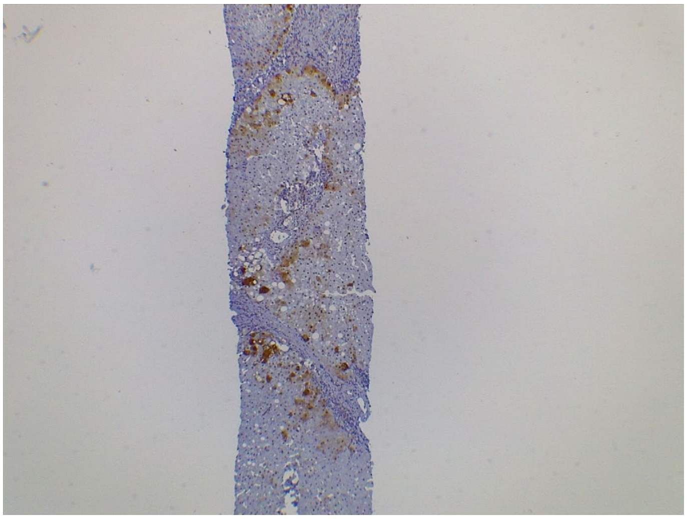

Patient 4 – NASH F4

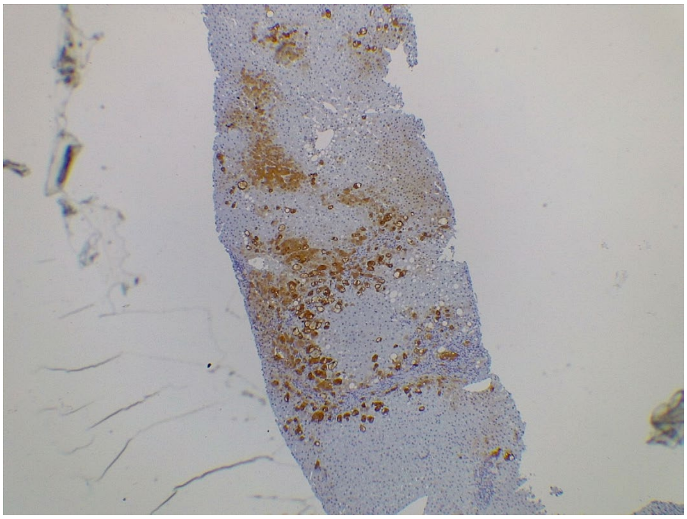

Patient 5 – NASH F4

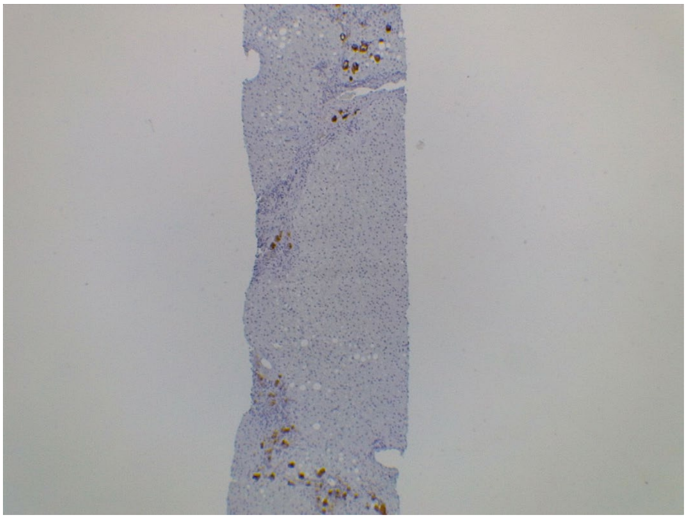

Patient 6 – NASH F3

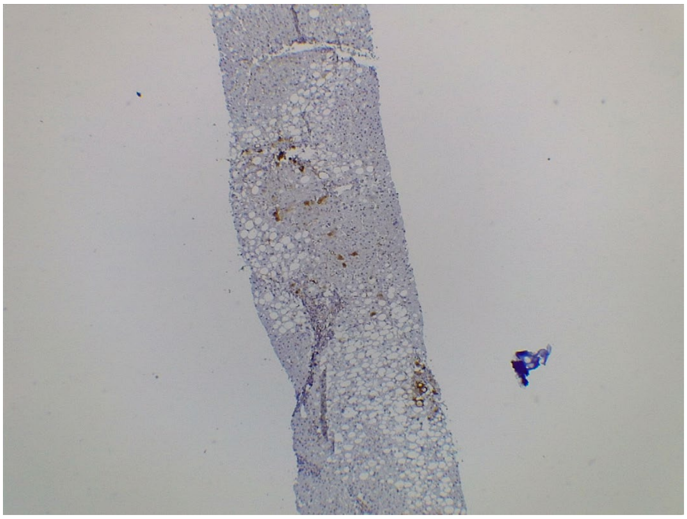

Source data file:
Overview immunohistochemical staining for AKR1B10 in 30 biopsies from patients with NAFLD.

Patient 7 – NASH F3

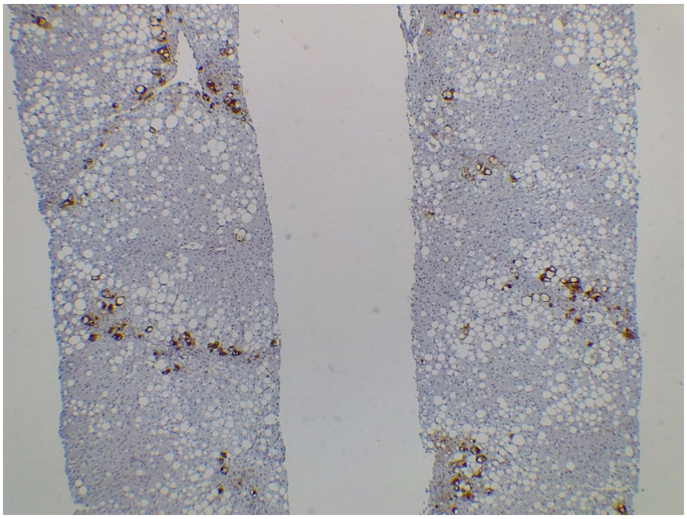

Patient 8 – NASH F3

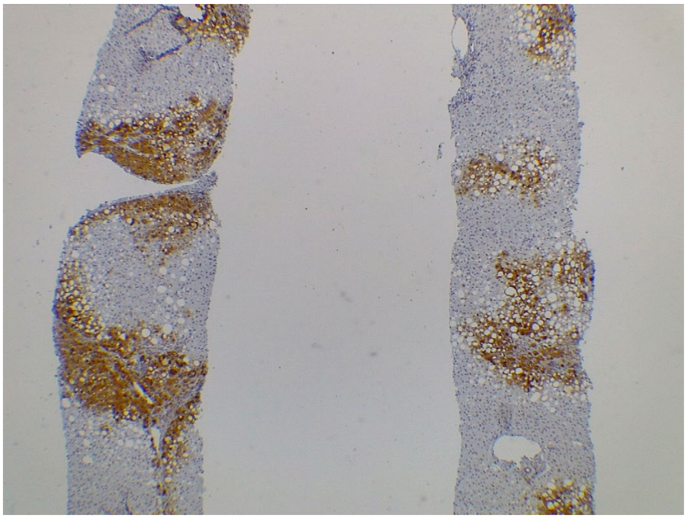

Patient 9 – NASH F3

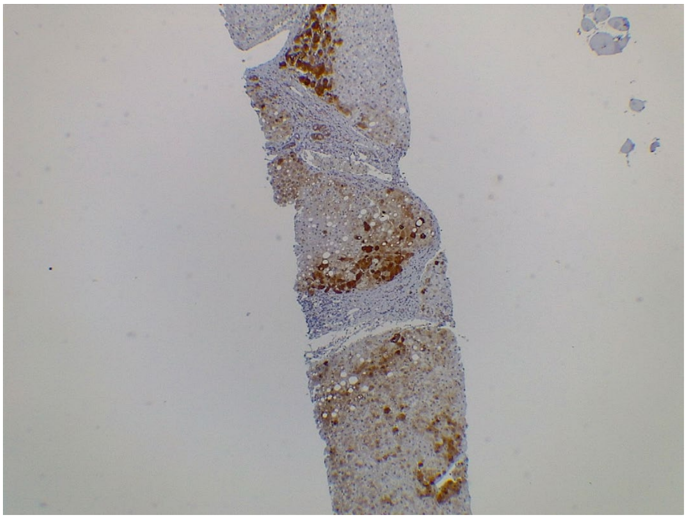

Patient 10 – NASH F3

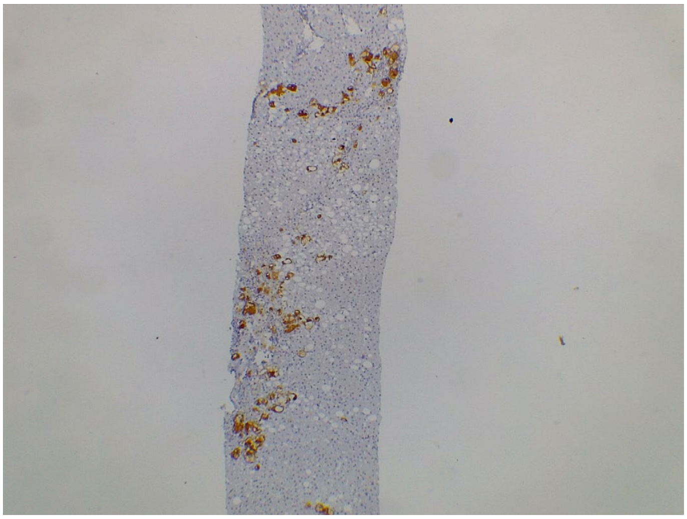

Patient 11 – NASH F3

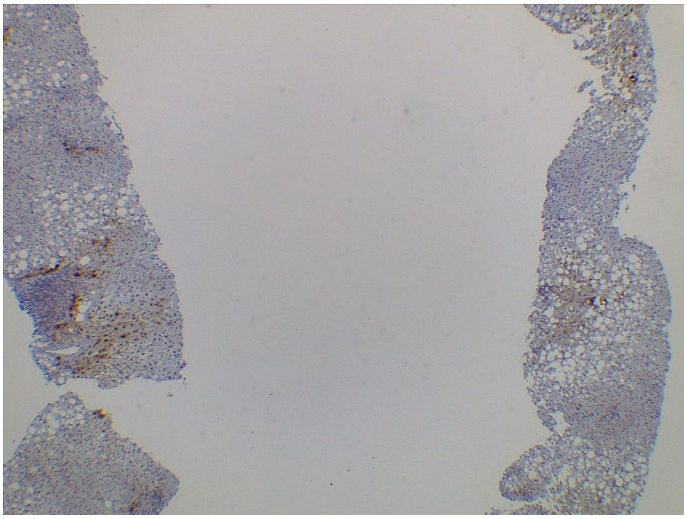

Patient 12 – NASH F3

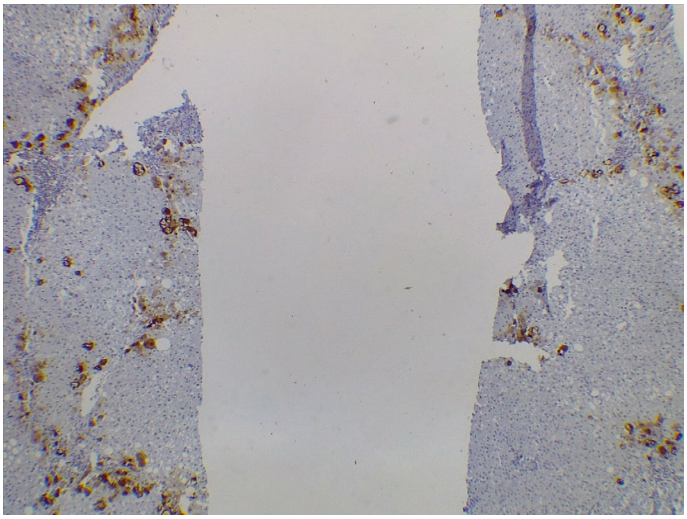

Source data file:
Overview immunohistochemical staining for AKR1B10 in 30 biopsies from patients with NAFLD.

Patient 13 – NASH F3

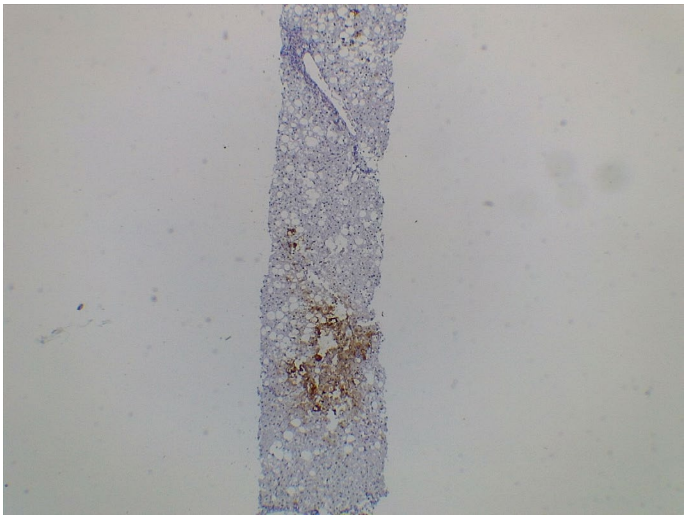

Patient 14 – NASH F2

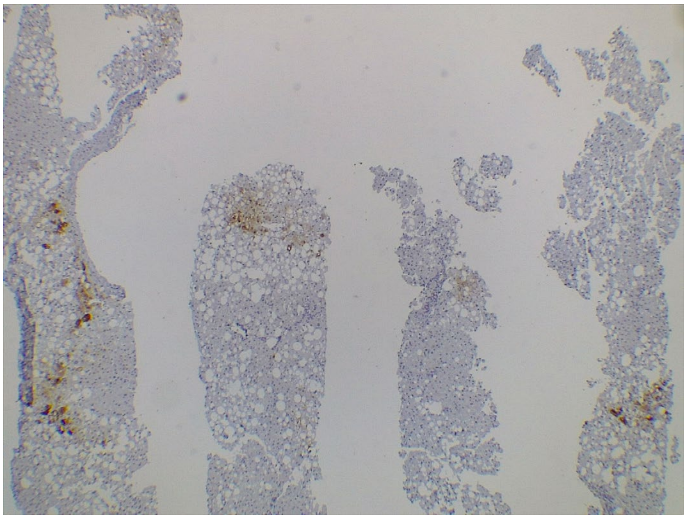

Patient 15 – NASH F2

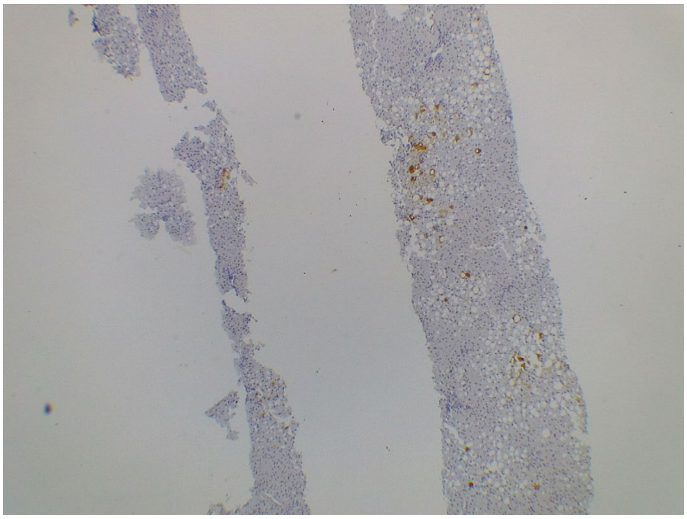

Patient 16 – NASH F2

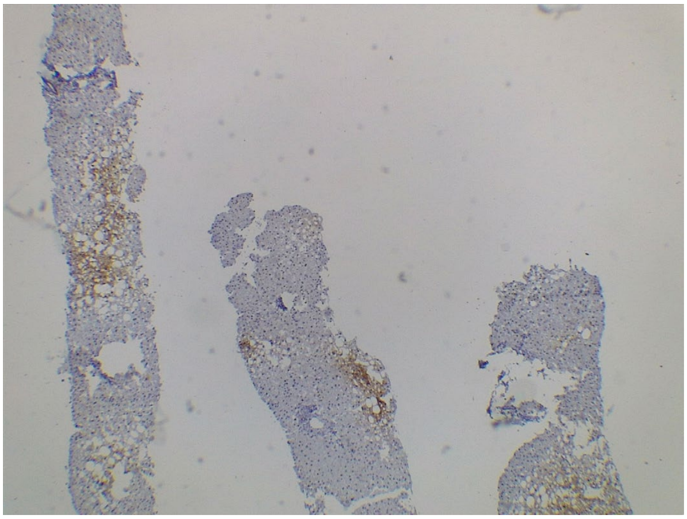

Patient 17 – NASH F2

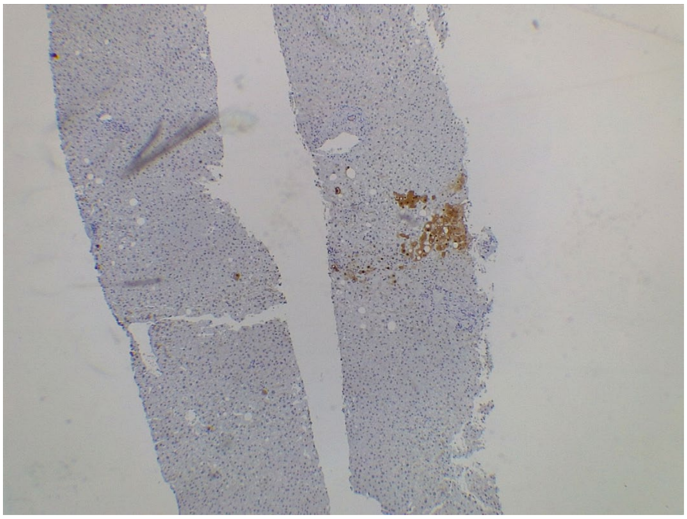

Patient 18 – NASH F2

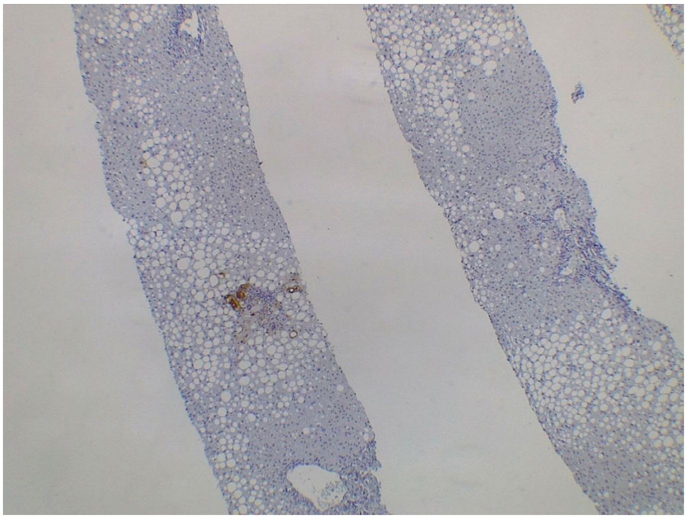

Source data file:
Overview immunohistochemical staining for AKR1B10 in 30 biopsies from patients with NAFLD.

Patient 19 – NASH F2

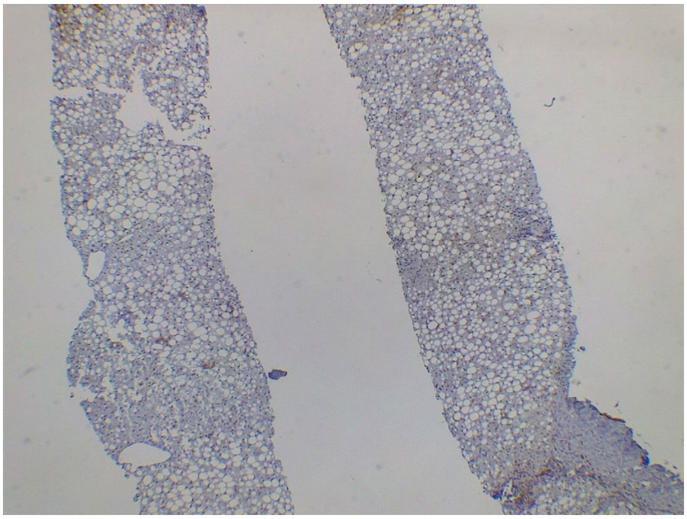

Patient 20 – NASH F0/1

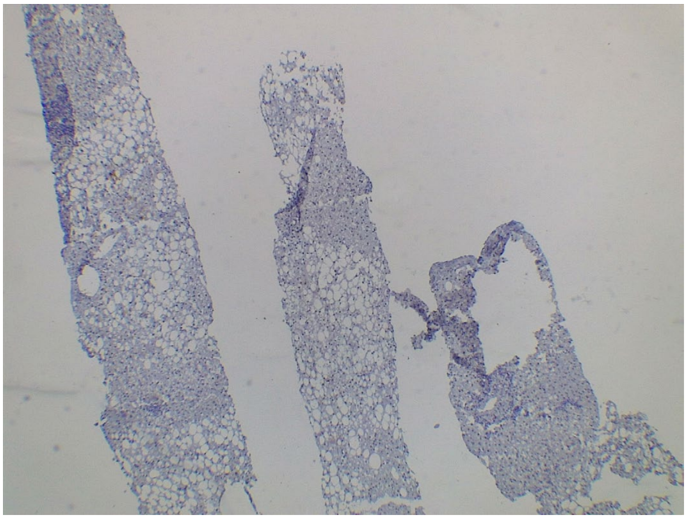

Patient 21 – NASH F0/1

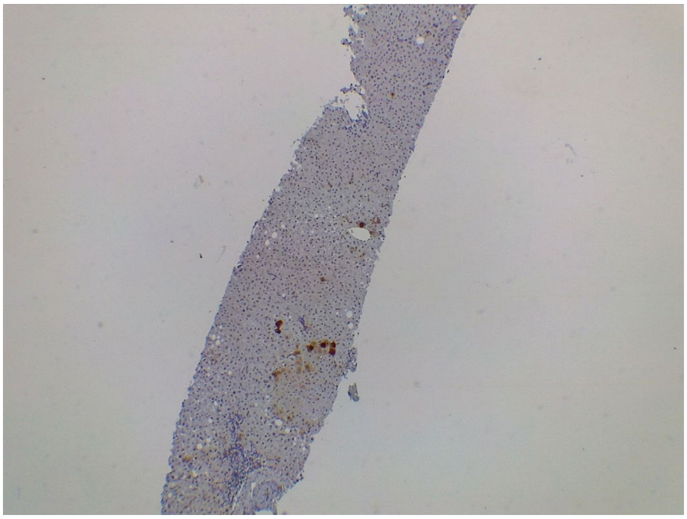

Patient 22 – NASH F0/1

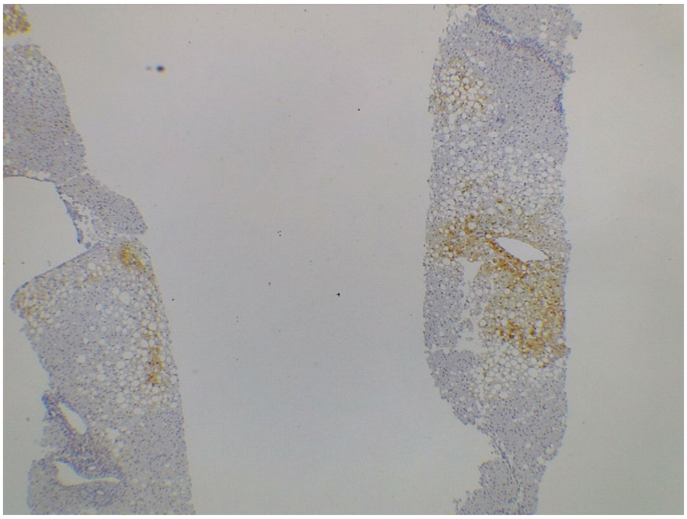

Patient 23 – NASH F0/1

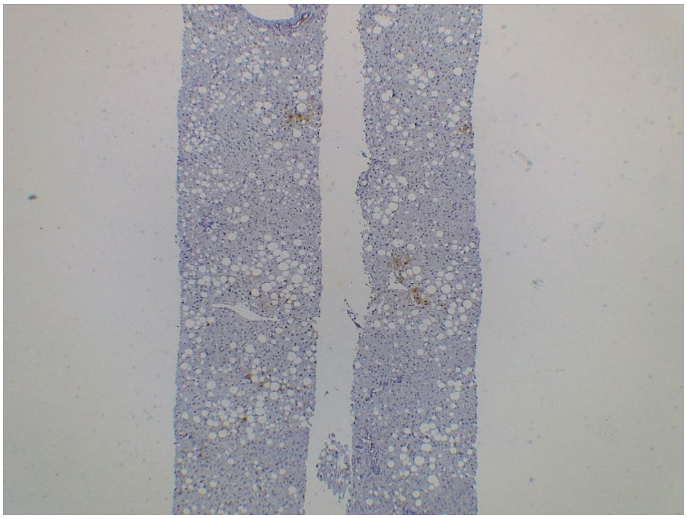

Patient 24 – NASH F0/1

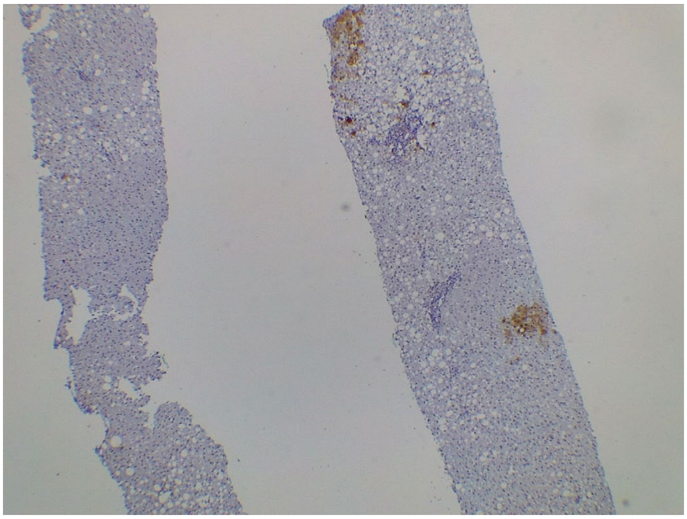

Source data file:
Overview immunohistochemical staining for AKR1B10 in 30 biopsies from patients with NAFLD.

Patient 25 – NAFL

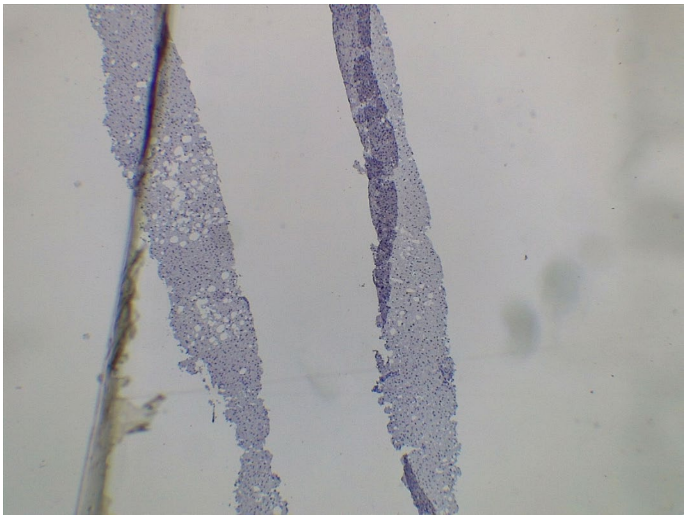

Patient 26 – NAFL

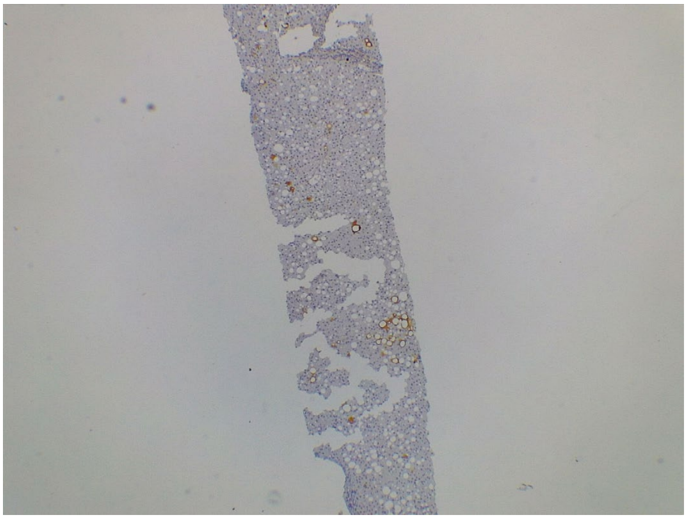

Patient 27 – NAFL

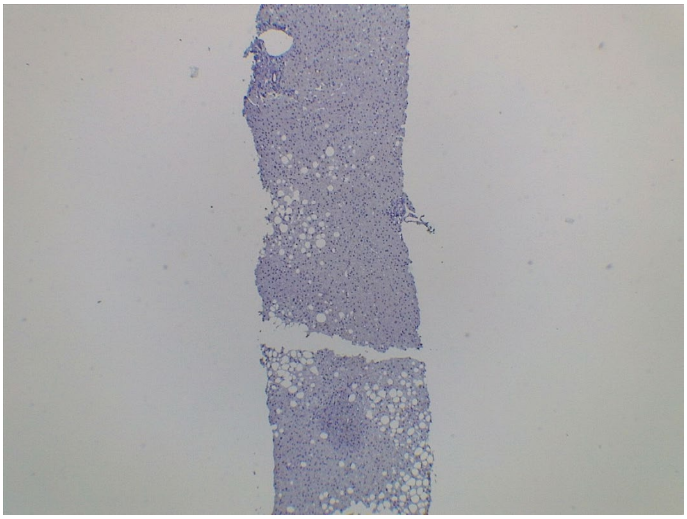

Patient 28 – NAFL

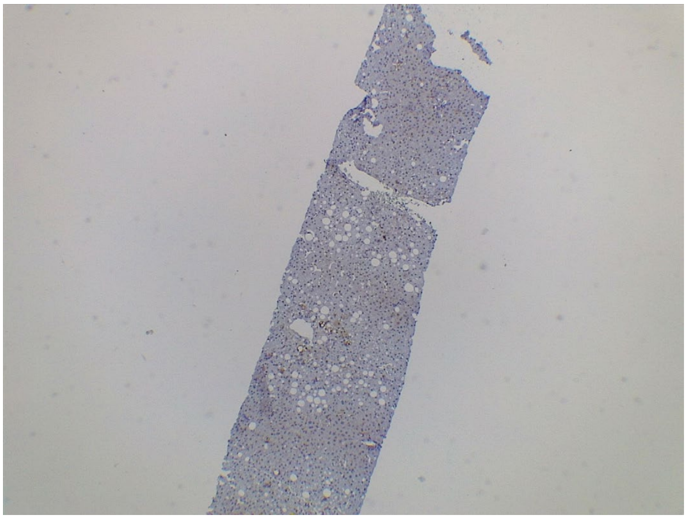

Patient 29 – NAFL

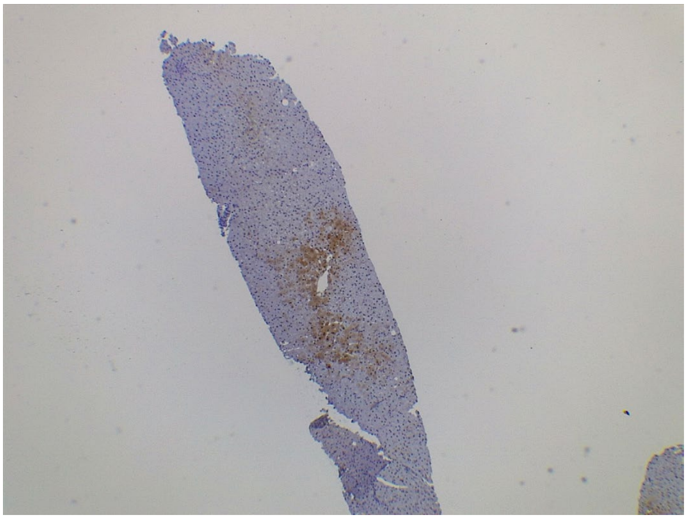

Patient 30 – NAFL

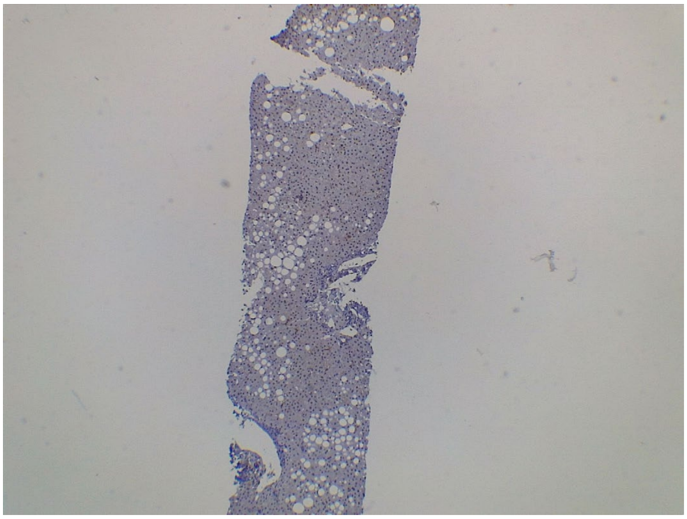

Supplement: Source Data Fig. 3 — Overview scans of the immunohistochemical stainings against AKR1B10 on 30 NAFLD biopsies. [file 42255_2023_775_MOESM4_ESM.pdf]
